# Modelling plant disease spread via high-resolution human mobility networks

**DOI:** 10.1098/rsif.2026.0213

**Published:** 2026-10-07

**Authors:** Varun K. Rao, Ryan Higgs, Hautahi Kingi, Filippo Radicchi, Santo Fortunato, Maria Litvinova

**Affiliations:** ^1^ Center for Complex Networks and Systems Research, Luddy School of Informatics, Computing, and Engineering, Indiana University, Bloomington, IN, USA; ^2^ Department of Epidemiology and Biostatistics, School of Public Health-Bloomington, Indiana University, Bloomington, IN, USA; ^3^ Onside, Christchurch, New Zealand

**Keywords:** mathematical modelling, networks, plant disease, disease spread, biosecurity, mobility network

## Abstract

Human mobility plays a crucial role in the spread of human diseases but is rarely quantified in plant disease epidemics. To address this gap, we integrate a unique, high-resolution network of human movements in New Zealand with a metapopulation model to mechanistically simulate pathogen transmission. We calibrate the model on the nationwide 2010 kiwifruit vine disease (Psa-V) outbreak and show that it reproduces the observed spatio-temporal spread, confirming that the human mobility network is a strong foundation for modelling human-mediated transmission dynamics. By analysing spatial infection trends, we find that most dispersal occurs locally, as often illustrated in the plant-outbreak literature. However, sporadic long-range connections are necessary to model a nationwide outbreak. Using the model as an *in silico* laboratory, we demonstrate that the severity of human-mediated pathogen transmission is highly sensitive to the timing and location of initial importation. We observe a potential causal link between seasonal labour patterns and epidemic risk in high-traffic seasons. This study showcases a novel data-driven framework for modelling the spatio-temporal spread of agricultural pathogens when human-mediated dispersal is epidemiologically relevant, underscoring the importance of leveraging human mobility networks for building better biosecurity systems.

## 1. Introduction

Plant diseases can devastate countries' economies, ecosystems and population health [1–6]. The loss and degradation of crop yields caused by these diseases have the potential to be catastrophic, as illustrated by the potato blight behind the Great Famine in Ireland in 1845–1850 [2]. Currently, the annual loss of crop yields owing to plant disease is estimated to be 30% globally [7], resulting in food insecurity and increasing threats to biosecurity across the globe. Based on a review conducted by Fielder *et al.* [8], at least 38 new plant pathogens were identified in new locations or on new hosts, while 10 global plant outbreaks in 2023 challenged crop production, food prices and food security.

Predicting the dynamics of plant disease outbreaks, and consequently responding to these persistent threats, is becoming increasingly complex [1] owing to changes in pathogens, their hosts, climate and human behaviours. Because plant pathogens spread through a wide range of vectors and dispersal mechanisms—most commonly insects, wind and rain—studies have emphasized environment-related host landscape modelling.

A variety of approaches have been utilized to understand and simulate the dispersal of the pathogen across the host landscape, including lattice-based models using kernel functions for dispersal [9–17], Bayesian methods [18,19], metapopulation modelling [15,20] and mean-field approximations [21]. For pathogens, especially fungi, dispersed through wind on long distances and capable of long-time survival, computational models employing atmospheric data and wind matrices [22–27] have been used to simulate the long-distance dispersal of the pathogen across the host landscape. For example, Radici *et al.* [28] combined short-range lattice-based landscape dispersal with atmospheric interconnectivity wind data to simulate how brown rot of peach could spread nationally within France. Some of the studies have also incorporated human-mediated dispersal together with environmental spread, either through a dispersal kernel function [29,30] or through a sampling from a distribution [3], when epidemiological data were available for calibration. With growing connectivity, human-mediated spread through contaminated equipment and transport [31–33], vector dispersal [30], infected plant material [34] or trade and supply networks [35–40] has become an important force in driving rapid, medium- and long-distance transmission events. The movement of plant material has been documented to connect potentially susceptible properties up to 350 km away from each other [41]. However, few studies have explored the impact of fluctuations in human mobility and consequently human-mediated dispersal on the long-distance spread of plant pathogens.

The few existing studies on the role of between-property mobility networks focus on trading [36–38], transport networks [39,40] or geographical distance [42,43]. However, these studies use static networks to model pathogen dispersal and do not account for the temporal changes in the magnitude of the traffic between the properties. Using inspiration from studies modelling spreading processes on temporal networks of livestock movements, we represent snapshots of movements at different time periods [44–47] to fill this gap. We propose a multi-scale metapopulation model based on empirical network data of human mobility between agricultural properties. Our approach combines a fully mixed population model for within-property spread and a mobility-based network model for between-property spread.

Our work eliminates the main barrier to integrating human mobility into plant disease epidemiology: the lack of high-quality spatio-temporal data suitable for plant disease modelling. Our unique dataset for the horticulture industry of New Zealand provides time-stamped origin–destination data for movements between properties. The data were provided and managed by Onside, an agritech company with biosecurity technology covering more than 20 000 properties from multiple industries across several countries, including New Zealand and Australia.

We leverage a susceptible–infected metapopulation model [48] to describe plant disease dynamics and calibrate the model to replicate the timing and severity of a past outbreak of *Pseudomonas syringae pv. actinidiae* (Psa-V) [49], a bacterial disease that was introduced to New Zealand in 2010 and is still present and widespread throughout the country. The disease devastated the country's kiwifruit industry [49–51], and was projected to cost kiwifruit growers $310 to $410 million for 5 years after the outbreak [52] owing to a combination of fast spread and pathogen severity [53,54]. Psa-V bacteria can colonize both inner and outer tissues of the kiwi from flowers to roots and grow best in humid conditions (e.g. after rainfall) in temperatures between 10 and 20${}^{\circ }$C. The bacteria can be spread through natural dispersal (mainly rain-splash and wind-blown rain) and human-mediated dispersal through the movement of infected materials, use of the same machinery and tools, and through vehicle tyres [32,53]. The bacteria can even survive on wet raincoats and wet soil on animal feet. While the pathogen can also survive on leaves for several weeks, depending on a combination of climatic factors [55], it is unknown how long and how often the bacteria can survive and remain infectious when transported by wind. Thus, local spread of Psa-V is thought to occur from a combination of environmental and human-mediated dispersal, while long-distance dispersal (>10 km) is primarily driven by human-mediated dispersal [53].

As human-mediated dispersal plays a role in both short- and long-range spread of Psa-V, we illustrate how our modelling approach can be utilized for Psa-V outbreaks using data-driven mobility networks. To incorporate the duality of the localized versus long-distance dispersal, we calibrate the model using within-property transmission probability that incorporates both environmental and human-mediated dispersal, and between-property transmission probability largely reflecting human-mediated spread. We explore three different temporal aggregations of the network, similar to those of Bajardi *et al.* [44], to reflect horticulture industry seasonality. By using this model incorporating seasonal dynamics of the human mobility network, we illustrate that the month of pathogen importation affects epidemic size, peak and onset time, demonstrating a direct link between movement seasonality and outbreak dynamics. Our modelling framework demonstrates how human mobility can be incorporated into a computational model to study the spread of plant disease and how the dynamics of human mobility can contribute to the long-distance dispersal of plant disease.

## 2. Methods

### 2.1. Movement data

We rely on data recorded by the Onside mobile app used by farmers and businesses servicing rural properties, such as contractors and other service providers. Data include the information when users enter (‘check-in’) one of the registered properties. Check-in events can be recorded either manually (i.e. a user checks in on arriving at a property) or automatically (i.e. triggered based on digital geofence technology) and are associated with time stamps on a minute resolution.

Onside data contain information about individual properties, such as their area and geographical coordinates, and the industry of the property: horticulture, agriculture and farming. Aiming to model the Psa-V outbreak in New Zealand, we focus on the N=2281 properties in the horticulture industry. A geographical map of the horticultural system in New Zealand reconstructed using Onside data is visualized in figure 1. According to the company statistics, the dataset represents approximately 70% of the kiwifruit production area in New Zealand and covers all the geographic areas where orchards exist.

**Figure 1.**
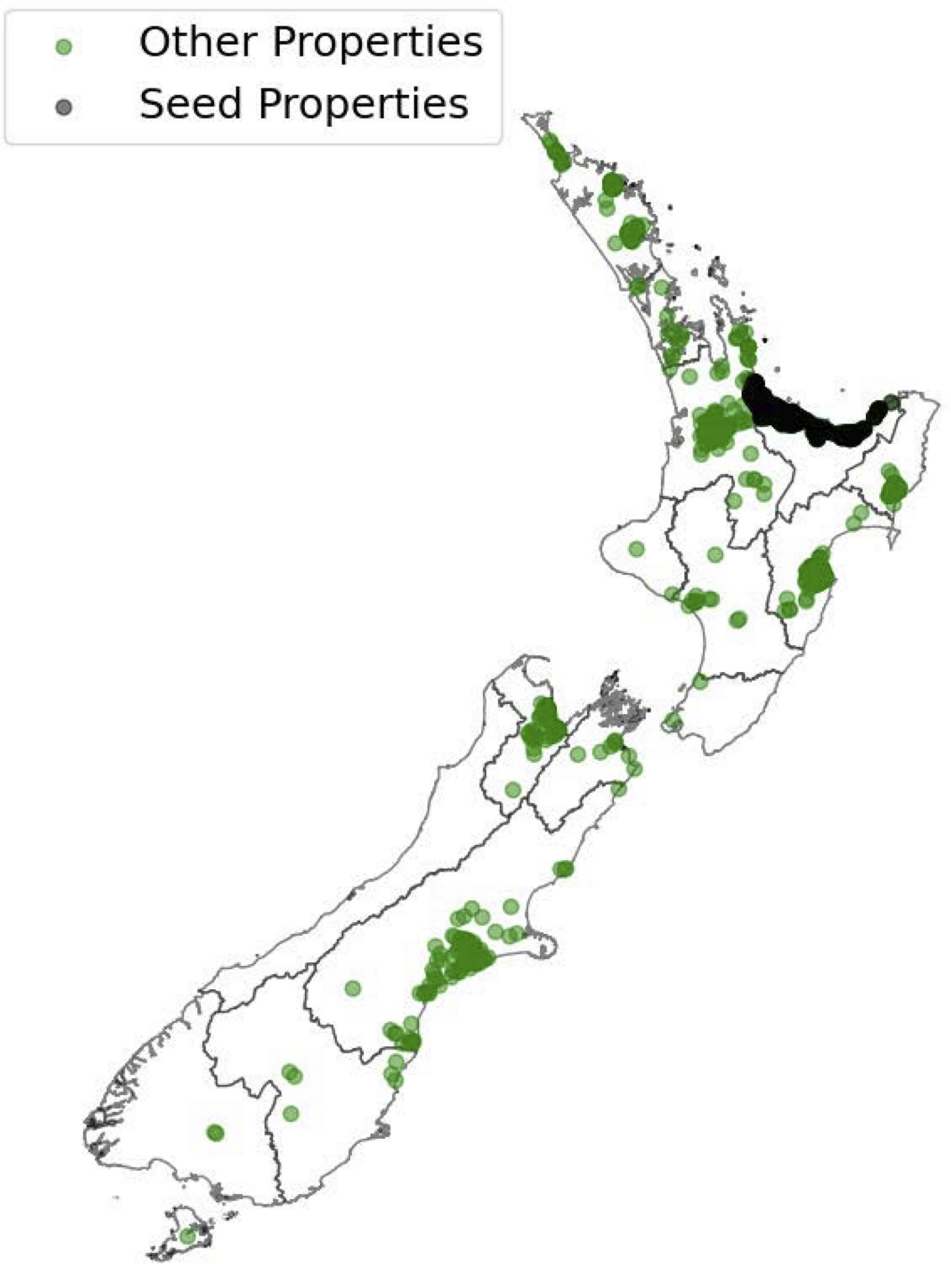
Map of the horticulture industry in New Zealand. Each point represents a property in the Onside dataset (N=2281 total properties). Black points denote the S=1300 properties located in the Bay of Plenty, which are used as seeds for the spreading dynamics simulated by our model.

From the Onside data, we extract all P=86624 movements recorded between 1 January 2022 and 31 December 2022 (see figure 2 for a schematic illustration). The data were preprocessed so time stamps are mapped to calendar months to match available epidemiologic monthly data. As a result, the same movement can appear multiple times in the data. Specifically, the pth movement data are denoted by $z_{p}=(o_{p},d_{p},m_{p})$, representing a person checking into property $d_{p}$ in month $m_{p}$, with $o_{p}$ representing the origin property the same person checked into previously.

**Figure 2.**
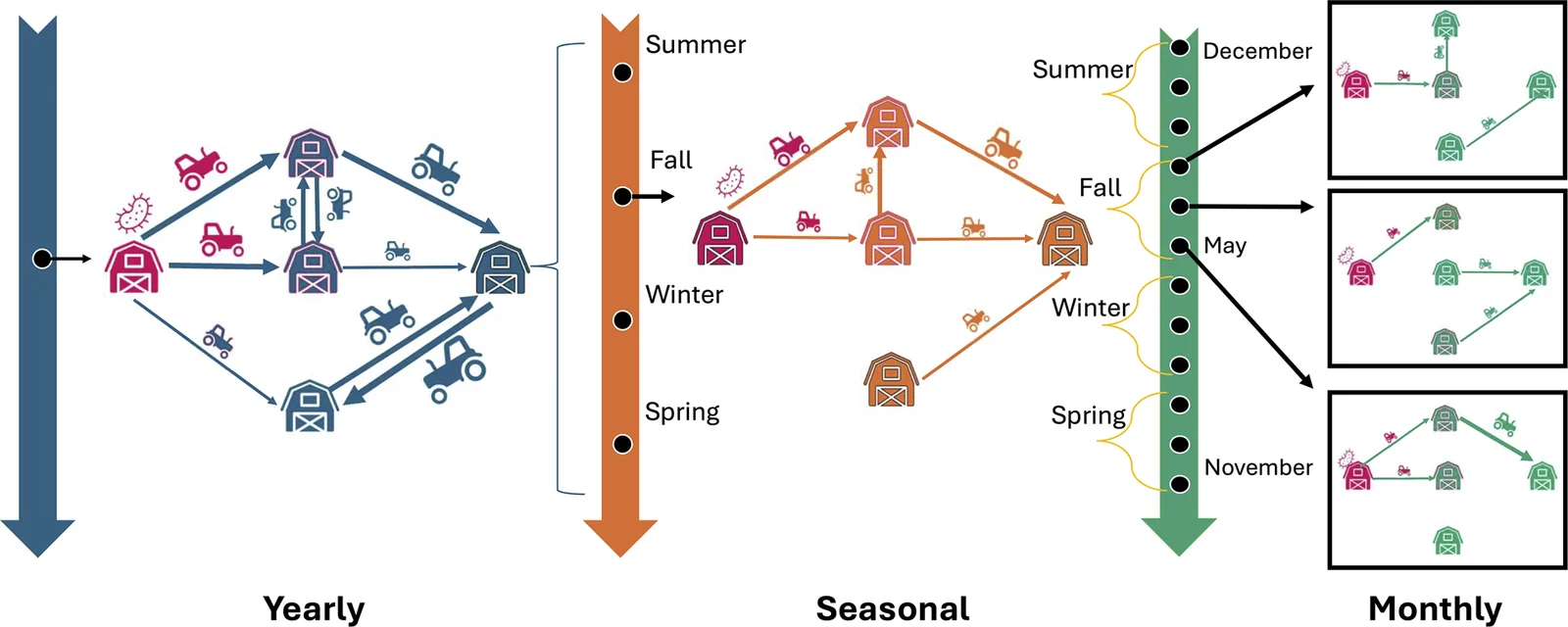
Schematic representation of mobility networks. Using the Onside movement dataset, we generate networks representing movements of workers between properties. In all networks, we measure the number of movements of people between properties. A movement is directed in nature and is recorded when a person leaves a source property and checks into a destination property. In this figure, a property is represented by a barn, and a movement between two properties is denoted by an arrow. The size of the tractor on top of an arrow is representative for the number of movements: the larger the tractor, the more movements occur. In a similar fashion, the width of the arrows also varies according to the number of movements. Depending on the aggregation level of the data, we define either yearly, seasonal or monthly networks. People moving between properties spread plant disease, and a higher frequency of movements between properties increases the probability of spreading. We note that starting the infection during different seasons or months can lead to different epidemic outcomes, since monthly movement networks differ. Infections are coloured red showing how seeding infections during different seasons or months can lead to different epidemic pathways.

We construct directed and weighted networks of movements for each month, excluding all movements with the same origin and destination. The generic element of the weight matrix for month m is

(2.1) $$\omega_{j\to i}^{\left(m\right)}=\frac{1}{30\;\text{days}}\;\sum_{p=1}^{P}\delta_{o_{p},j}\delta_{d_{p},i}\delta_{m_{p},m},\;$$

where the Kronecker delta function is such that $\delta_{x,y}=1$ if x=y and $\delta_{x,y}=0$ otherwise. $\omega_{j\to i}^{\left(m\right)}$ quantifies the average number of daily movements from property j to property i observed in month m.

Similarly, we can generate directed weighted networks of movements for the four seasons: autumn, winter, spring and summer. In such a case, the generic element of the weight matrix is

(2.2) $$\omega_{j\to i}^{\left(s\right)}=\frac{1}{90\;\text{days}}\;\sum_{p=1}^{P}\delta_{o_{p},j}\delta_{d_{p},i}\delta_{f(m_{p}),s},\;$$

where f(m) is the function that maps months to seasons, defined as follows: f(m)= autumn for m= March, April and May, f(m)= winter for m= June, July and August, f(m)= spring for m= September, October and November and f(m)= summer for m= December, January and February.

The yearly version of the network is instead given as

(2.3) $$\omega_{j\to i}=\frac{1}{365\;\text{days}}\;\sum_{p=1}^{P}\delta_{o_{p},j}\delta_{d_{p},i},\;$$

where all movements in the year are aggregated together.

In table 1, we show how the level of aggregation affects some basic network metrics using the connected nodes (properties having at least one non-zero-weight incoming or outgoing edge). We calculate the average values over all graphs for each temporal aggregation for the number of nodes ⟨N⟩, the number of edges ⟨E⟩, the first moment of the edges' weights ⟨ω⟩ and the second moment of the edges' weights $\left\langle \omega^{2}\right\rangle$.

**Table 1.** Network statistics. For each level of data aggregation, we report the corresponding average number of nodes ⟨N⟩, average number of edges ⟨E⟩, average value of the edges' weights ⟨ω⟩ (measured in number of daily movements) and the second moment of the edge weight $\left\langle \omega^{2}\right\rangle$.

aggregation	**⟨N⟩**	⟨E⟩	**⟨ω⟩**	**$\left\langle \omega^{2}\right\rangle$**
yearly	2281	23418	0.01	0.84
seasonal	1627	8124	0.03	0.80
monthly	1153	3413	0.07	0.86

By definition, the yearly network contains more nodes and edges than the monthly and seasonal networks. However, the weight of the connections is greater in the monthly and seasonal networks. This aligns with an empirical understanding of how temporal networks exhibit ‘bursty’ behaviour with large amounts of activity occurring in a short period of time, followed by periods of inactivity [58]. Estimates of the second moment highlight how, at all levels of aggregation, there is large heterogeneity in the weight distribution across the edges.

### 2.2. Metapopulation model

We are interested in developing a model for the spread of Psa-V that is calibrated on the monthly number of infected properties between February 2011 and March 2012 [52]. To this end, we make the assumption that the 2022 Onside data at our disposal can be used to generate movement networks representative of 2011 and 2012. Specifically, we define the weight of the directed connection j→i at time t as

(2.4) $$w_{j\to i}(t)=\left\{ \begin{array}{ll} \omega_{j\to i}^{\left(m=g(t)\right)} & \text{monthly resolution}\\ \omega_{j\to i}^{\left(s=f(g(t))\right)} & \text{seasonal resolution}\\ \omega_{j\to i} & \text{yearly resolution}. \end{array}\right.$$

In the above expression, g(t) is the function that maps the model's time t to calendar months; f(m) maps calendar months to seasons (already used in equation (2.2)).

The proposed metapopulation model combines two spreading mechanisms: within-property and between-property. It utilizes a mean-field equation that approximates the level of infection that occurs within a property, given the within- and between-property forces of infection, which are defined by a specific parameter configuration. The resulting dynamics are deterministic in nature but incorporate the uncertainty associated with the variability in initial conditions, in particular the choice of the seed property. Each property i, composed of $H_{i}$ hectares of land, is represented as a population of susceptible and infected hectares. We denote the fraction of infected hectares on the property i at time t as $I_{i}(t)$ and the fraction of susceptible hectares for property i at time t as $S_{i}(t)$. By definition, we have that

$$S_{i}(t)+I_{i}(t)=1,\;\forall i\in 1,\ldots ,N\;\text{and}\;\forall t.$$

The level of transmission within a property is driven by $I_{i}(t)$ and $S_{i}(t)$ (within-property transmission), as well as the importation of infection from other connected properties (between-property transmission), and can be described as

(2.5) $$\begin{array}{ll} \frac{dI_{i}(t)}{dt}= & \beta_{w}I_{i}(t)S_{i}(t)\Theta \left[I_{i}(t)-I_{i}^{\min}\right]\\ & +\beta_{b}S_{i}(t)\sum_{j=1}^{N}w_{j\to i}(t)I_{j}(t)\Theta \left[I_{j}(t)-I_{j}^{\min}\right], \end{array}$$

for i=1,…,N. Θ(x) is the Heavyside function defined such that Θ(x)=1 if x>0 and Θ(x)=0 otherwise. In equation (2.5), the force of infection within a property is denoted as $\beta_{w}$, and the force of infection between properties is denoted as $\beta_{b}$. To be considered infected, property i must have a level of infection above the threshold $I_{i}^{\min}$, where $I_{i}^{\min}={\left(H_{i}\;625\;{\mathrm{plants}\;\mathrm{ha}}^{-1}\right)}^{-1}$; 625 is a realistic estimate of the average number of plants per hectare [59]. This threshold value is set to ensure that at least one plant on the property is infected before spreading occurs.

We assume that the mean-field approximation [60] in equation (2.5) describes the average dynamical behaviour of the system. We numerically integrate it using Newton's method (see supplementary material for details). The initial condition at time $t=t_{0}$ is such that $I_{i}(t_{0})=0$ for all i except for the seed property $s_{0}$ for which $I_{s_{0}}(t_{0})=0.1$. As shown in the supplementary material, the results of our analysis are robust to the variation of the initial level of infection.

It can be difficult to detect early-stage symptoms of plant disease, in part owing to less routine monitoring. Thus, we require that $I_{i}(t)>D$ for the property i to be counted as infected at time t. We define the cumulative number of infections at time t measured in our model as

(2.6) $$O(t)=\sum_{i=1}^{N}\Theta \left[I_{i}(t)-D\right].$$

We stress that the specific choice of the detection threshold D does not affect how spreading unfolds, but affects how infections are recorded to be compared with observed epidemic curves. Once the level of infection on a property i, $I_{i}$, exceeds the threshold value for D, the property is included in the number of cumulative infections, as seen in equation (2.6), and the transmission continues as previously described. Thus, D should be interpreted as an effective detection threshold, where the set parameter value combines the progress of the infection before the symptoms are detected and reported or before the surveillance detects infection, as well as biological incubation.

By solving the mean-field equations of the model, we estimate the expected number of properties that will be infected given a specific combination of values of $\beta_{w}$ and $\beta_{b}$, and a seed property $s_{0}$. While a value for D must be used in our model, it does not influence the results of the outbreak, just how it is observed.

### 2.3. Model calibration

We use the data on the outbreak dynamics from a 2012 report [52] relying on data by Kiwifruit Vine Health (KVH), a biosecurity organization established in December 2010 [49] and responsible for protecting the kiwifruit industry from pests and disease. As the KVH disaggregated data are not publicly available, we extract the monthly values from the reported aggregated curve using a plot digitizing tool [61]. The incidence curve shown in figure 3 details the observed total monthly infections from February 2011 to March 2012. Since infections had occurred before March 2011 [62] but were not available in the report, we exclude February from our calibration procedure. We also use the number of infected properties per growing season [62] to confirm the total number of infections in March 2012.

**Figure 3.**
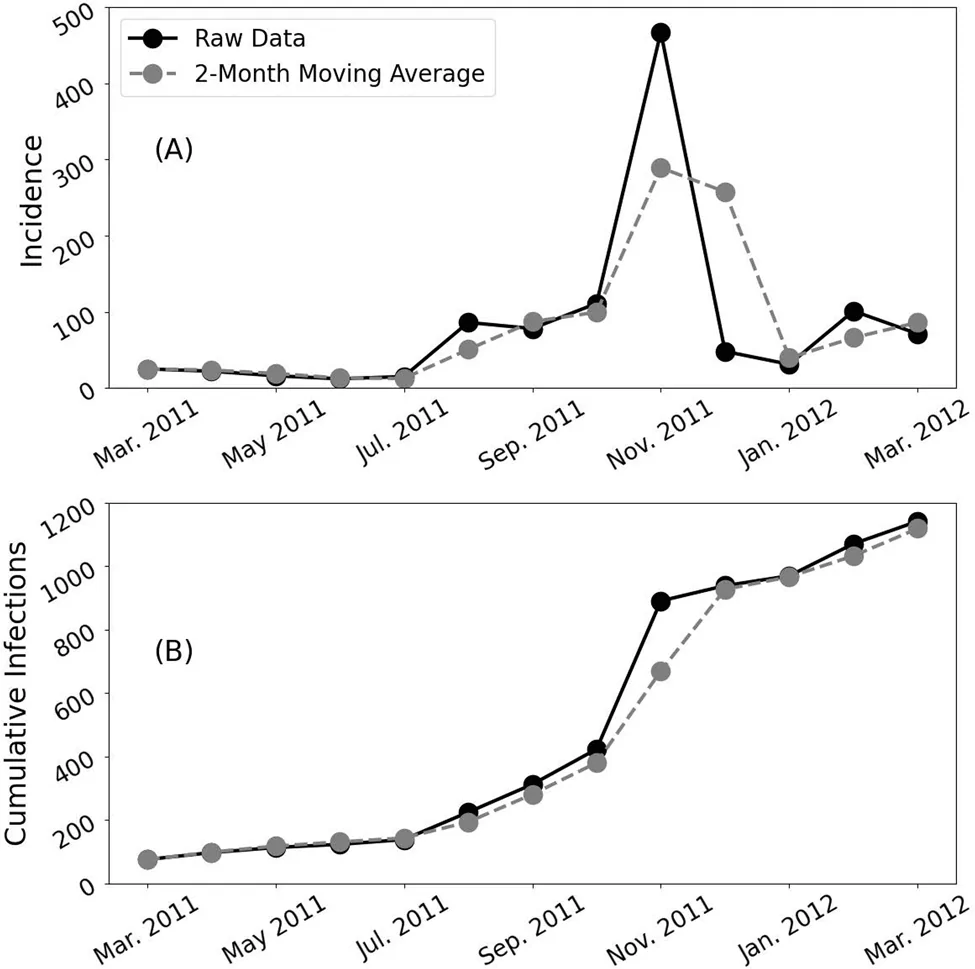
Dynamics of the Psa-V epidemic in New Zealand. (A) number of new monthly infected properties and (B) cumulative number of monthly infected properties. The solid curve in both panels represents the raw data extracted from the report written by Greer & Saunders [52]. In this, the monthly infection data start from February 2011 and end in 21 March 2012, but the actual outbreak started in November 2010 and has now become endemic in New Zealand. Thus, the leftmost point in the raw data corresponds to the number of properties that became infected between November 2010 and February 2011. The grey curve is a two-month moving average of the raw time series. The moving average is calculated starting from March 2011.

According to Rosanowski *et al.* [62], the largest spikes in infection occurred in the spring during the 2011/2012 growing season and the spring of the 2012/2013 growing season. Symptom emergence is greatest in the spring for Psa-V owing to the warm, humid conditions and increased rainfall [53]. Although Psa-V symptoms are visually apparent once established, some orchards may have been infected earlier but only reported later owing to biological latency, mild or ambiguous early symptoms, and limited awareness during the early outbreak phase. Given the potential impacts of the weather on the observed/reported infection incidence, we also estimate a two-month moving average as an alternative reference curve for calibration (supplementary material).

The data used in our calibration procedure contain 14 monthly counts of newly infected properties at the national level (not geolocalized). We denote this time series as $\vec{x}=(x_{1},x_{2},\ldots ,x_{14})$, with data points indexed in chronological order. This means that, for example, $x_{1}$ is the value of the incidence curve in February 2011, while $x_{14}$ refers to the incidence in March 2012. To compare the outcome of the metapopulation model with such real data, we discretize the time series of equation (2.6) by extracting a single value for each month from February 2011 to March 2012, i.e. $\vec{m}=(m_{1},\ldots ,m_{14})$. Each of these values corresponds to the cumulative number of infections measured at the end of the corresponding month. For example, $m_{1}$ is the cumulative number of infections measured in the model at midnight of 1 March 2011. From the time series describing the cumulative number of infections, we immediately derive the incidence time series as $y_{1}=m_{1}$ and $y_{c}=m_{c}-m_{c-1}$ for c=2,…,14.

The outcome of the metapopulation model depends on six tunable parameters (see table 2). In our calibration procedure, we assume that the importation occurs on 1 November 2010, effectively reducing the number of free parameters to five [52,62]. This assumption was made to accommodate the time necessary for the detection to occur, resulting in almost four months of cryptic, unreported transmission before the model outcome and raw data can be compared.

**Table 2.** Model parameters and their description.

parameter	description
R	temporal resolution for the movement network
$\beta_{w}$	within-property transmission rate
$\beta_{b}$	between-property transmission rate
$s_{0}$	initial seed property
$t_{0}$	initial time of the spreading process
D	detection threshold

For each level of temporal aggregation, we perform a grid search to find the best-fitting values for the other model parameters ($\beta_{w}$, $\beta_{b}$, D, $s_{0}$), which we detail in more depth in the supplementary material. Potential configurations are filtered using the heuristic criterion detailed in equation (2.7). We run separate calibrations for the different levels of temporal network aggregation. Also, based on the literature [52,62], we use seed nodes only from the Bay of Plenty (see figure 1), as the outbreak originated from that region. The goal of the calibration is to ensure that the resulting dynamics fit incidence data with an acceptable level of uncertainty, a common goal of infectious disease models [63].

Given a configuration θ of the free model parameters and an aggregation R, we numerically integrate equation (2.5) of the metapopulation model to generate the incidence curve $\vec{y}(\theta )=(y_{1}(\theta ),\ldots ,y_{14}(\theta ))$. Such a configuration is considered viable, i.e. θ∈V, if

(2.7) $$\left|x_{c}-y_{c}(\theta )|<\max\{k,bx_{c}\right\}$$

for all c=2,…,14. This allows us to select model parameter configurations whose outcome is compatible with the ground truth within the entire observation window for the data. In the supplementary material, we show the percentage of viable configurations for each combination of k and b. We chose k=30 and b=0.8 as they were the first values with more than 1000 viable configurations for all network aggregations for the raw data. We use this criterion to prevent overfitting to any one specific month and filter configurations that exhibit growth trends not compatible with the incidence data. The best configuration $\hat{\theta }$ is defined as the one minimizing the root mean squared error (RMSE), i.e.

(2.8) $$\hat{\theta }=\arg\min_{\theta \in V}\sqrt{\frac{\sum_{c=2}^{14}(x_{c}-y_{c}(\theta ){)}^{2}}{13}}.$$

Note that in both equations (2.7) and (2.8), we start from the index c=2, thus excluding the first data point in the time series. This same procedure is also used for the best fit using the two-month moving average (table 3).

**Table 3.** Best-fitting parameter values for each temporal aggregation using the raw and moving average incidence data. We use bold font to highlight the best-fitting aggregation level for each type of reference curve.

data	aggregation	$\beta_{b}$	$\beta_{w}$	D
raw	monthly	0.03	0.09	0.4
	**seasonal**	0.03	0.08	0.8
	yearly	0.04	0.07	0.8
moving average	**monthly**	0.02	0.10	0.4
	seasonal	0.03	0.07	0.8
	yearly	0.02	0.10	0.4

### 2.4. Spatio-temporal characterization of the spreading dynamics

Our metapopulation model of equation (2.5) allows us to keep track of the level of infection $I_{i}(t)$ of each property i at time t. Similarly to what was described in the previous section, we discretize such a continuous time series into 14 values $\vec{m}=(m_{1}^{\left(i\right)},\ldots ,m_{14}^{\left(i\right)})$, each value corresponding to the months between February 2011 and March 2012. We define the time to detect infection for property i, namely $c_{i}^{*}$, as the minimum value of the index c for which $m_{c}^{\left(i\right)}>D$. We finally measure the monthly detection distance $d_{i}^{*}$ between i and the closest property j such that $c_{j}^{*}=c_{i}^{*}-1$. Since multiple properties can be detected at the same time $c^{*}$, we compute the minimum, mean and maximum of the quantity $d^{*}$ for all properties that are first detected as infected at time $c^{*}$.

Similarly, we estimate the distance between property i, first infected at time $c_{i}^{*}$, and each of the connected properties j that are first infected at time $c_{j}^{*}=c_{i}^{*}-1$, further referred to as the contact distance $d_{i,j}$. As multiple $d_{i,j}$ may be observed for each value $c^{*}$, we compute the minimum, mean and maximum of $d_{i,j}$ for all properties that are first detected as infected at $c^{*}$.

These distance metrics allow us to track whether the outbreak distance changes over time. The contact distance shows the distance between infectees and their infectors, while the outbreak distance shows the distance between incident infections, providing information on how the front of the epidemic expands month by month.

## 3. Results

### 3.1. Replicating observed spreading dynamics

In table 3, we report the values of the best estimates of our model grouped by the level of data aggregation. We display separate results depending on whether the reference time series is the raw incidence data or their moving average. Two aspects become apparent. First, the parameter estimates are robust across the various levels of aggregation, with the within-property contribution being about three times larger than its between-property counterpart. Second, their posterior distributions are well-peaked around their mode (supplementary material).

We visually compare the incidence curves from the raw data and the model estimates corresponding to the best fit in figure 4A. The bands for each of the model's curves represent the uncertainty of the incidence estimates owing to the choice of the seed $s_{0}$ when using the best-fitting parameter values for $\beta_{w}\text{,}\;\beta_{b}\;\text{and}\;D$, given the temporal aggregation and incidence curve. The uncertainty is calculated as the range (the difference between the maximum and minimum values) of monthly incidence across all viable seed properties for the best-fitting epidemiological parameters ($\beta_{b},\beta_{w},D$). The viable seeds are chosen according to equation (2.7). Within the insets of figure 4, we compute the total percentage of viable configurations and the percentage of unique seeds observed given the best-fitting values for $\beta_{b},\beta_{w}$ and D for each temporal aggregation. The viable configurations represent the *total* number of configurations of $\beta_{b},\beta_{w},D\;\text{and}\;s_{0}$ that successfully pass our filtering, while the percentage of best-fitting seeds denotes the number of unique seeds when the values of $\beta_{b},\beta_{w}\;\text{and}\;D$ are set to their best-fitting value, telling us the fraction of plausible seed properties within the network. In figure 4B, the model is fitted using the two-month moving average of the incidence curve. In the supplementary material, we show the individual trajectories within the uncertainty bands for each network aggregation and by data type (supplementary material, figure S11).

**Figure 4.**
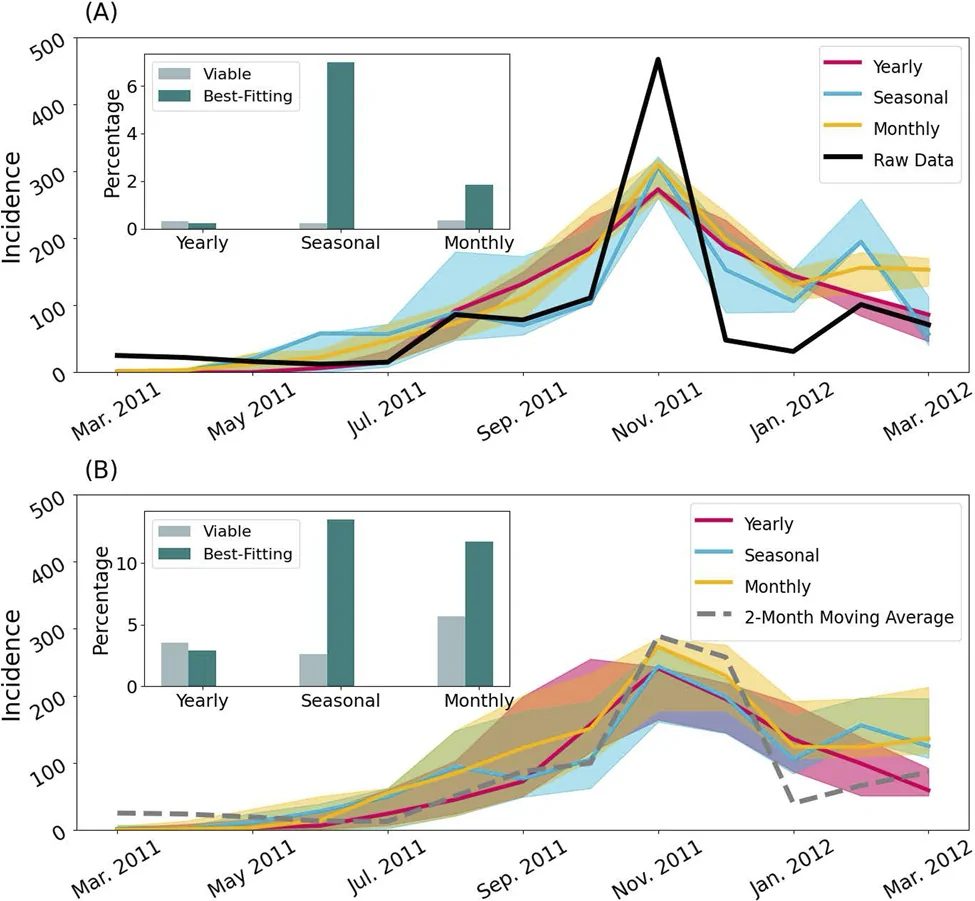
Best-fitting epidemic models. (A) We plot the incidence time series obtained from the epidemic model when setting its parameter values to their corresponding best fits. Different colours refer to different levels of temporal aggregation of the movements between properties: yearly (magenta), seasonal (blue) and monthly (orange). The shaded areas define the uncertainty associated with the selection of the seed properties, computed as the range of the incidence values measured over the viable seeds for the best-fitting values for $\beta_{b},\beta_{w}\;\text{and}\;D$. Model's curves are compared against raw data (black). The inset displays two different statistics: in grey, we show the percentage of all viable configurations for each aggregation level; in teal, we show the percentage of all unique seeds that are viable when holding $\beta_{b},\beta_{w}\;\text{and}\;D$ to their best-fitting value. This analysis was conducted for the various levels of temporal aggregation that can be in the model. (B) Same as in (A) but obtained when fitting the model against the two-month moving average incidence curve (grey dashed).

The results shown in figure 4 illustrate that the model can capture the timing of epidemic dynamics well for all levels of temporal aggregation but underestimates the peak incidence in all aggregations for raw data. The yearly aggregation underestimates the peak by 41.5%, the seasonal aggregation by 34.3% and the monthly aggregation by 33.8%. However, the magnitude of this underestimation is the characteristic of monthly data cadence and may change with higher temporal resolution. When the model is calibrated to fit the two-month moving average of the incidence curve, it fits the epidemic dynamics better compared to the raw data. This can also be observed as the underestimation of the peak incidence decreases for the moving averages, with the yearly aggregation resulting in a 16.6% lower incidence, the seasonal aggregation 15.6% and the monthly aggregation 5.53%. These are all smaller underestimations than what was seen when calibrating to the raw data. This may be attributed to factors not incorporated in the model, such as weather impacts on the transmission probability and symptoms, improvements in surveillance and changes in interventions and industry practices, not connected with human mobility. Moreover, the difficulty of fitting the raw peak incidence well arises from the extreme steepness of the outbreak peak, with a doubling time being less than the available data time step (one month). This also highlights the need for higher temporal disaggregation of infection data for calibration using the proposed modelling approach when highly transmissible pathogens are analysed.

Of all levels of temporal aggregations, the seasonal configuration provides the best fit, i.e. the smallest RMSE (equation (2.8)), for the dynamics of the observed incidence (figure 4A). It also has the largest percentage of best-fitting configurations, but the smallest percentage of viable seeds. For the two-month moving average (figure 4B), the monthly network provides the best fit. Compared to the raw incidence, the percentage of viable seeds and best-fitting configurations is greater than for the moving average across temporal aggregations. The RMSE for the best-fitting configurations is smaller for the moving average compared to the raw data. This indicates that the model struggles less with smoother dynamics when doubling time is comparable to, or preferably shorter than, the temporal observational scale.

The model is well calibrated in terms of outbreak timing, with the correct peak month being captured by all levels of temporal aggregations, for both the raw data and its moving average. Additionally, the best-fitting seasonal model calibrated for raw incidence captures not only the peak that occurs in November 2011 but also the second incidence increase that occurs in February 2012.

### 3.2. Spatial spread

As seen in figure 5, most spreading events are initially local. Over 90% of all newly infected properties are less than 10 km from their closest infectors (supplementary material), illustrating the dominance of local spread. However, rare long-distance events pull the average distance between newly infected properties and the closest infected property into the range of 10–100 km (figure 5). This feature is a direct result of the movement network's spatial structure, with most movements (71.3%) being short range (<10 km), 12.2% of movements being >20 km and rare cases reaching 1000 km (the complementary cumulative distribution function of the movement distribution is shown in the supplementary material). The importance of such movements for transmission increases as the outbreak continues, with the average distance from the closest infected property consistently increasing each month. The heterogeneity of the distance between potential infectors and newly infected nodes also increases with time owing to two factors: (i) the number of close susceptible nodes decreases and (ii) the pathogen reaches close but less connected properties (a smaller number of movements). This leads to an initially small and local outbreak spreading far beyond its origin. In the supplementary material, we show how the cumulative infection distance from the initial seed grows over time, spreading more than 1000 km from where it is seeded. We observe that growth from the seed occurs in a staggered manner, with an increase in distance from the seed property every two to three months.

**Figure 5.**
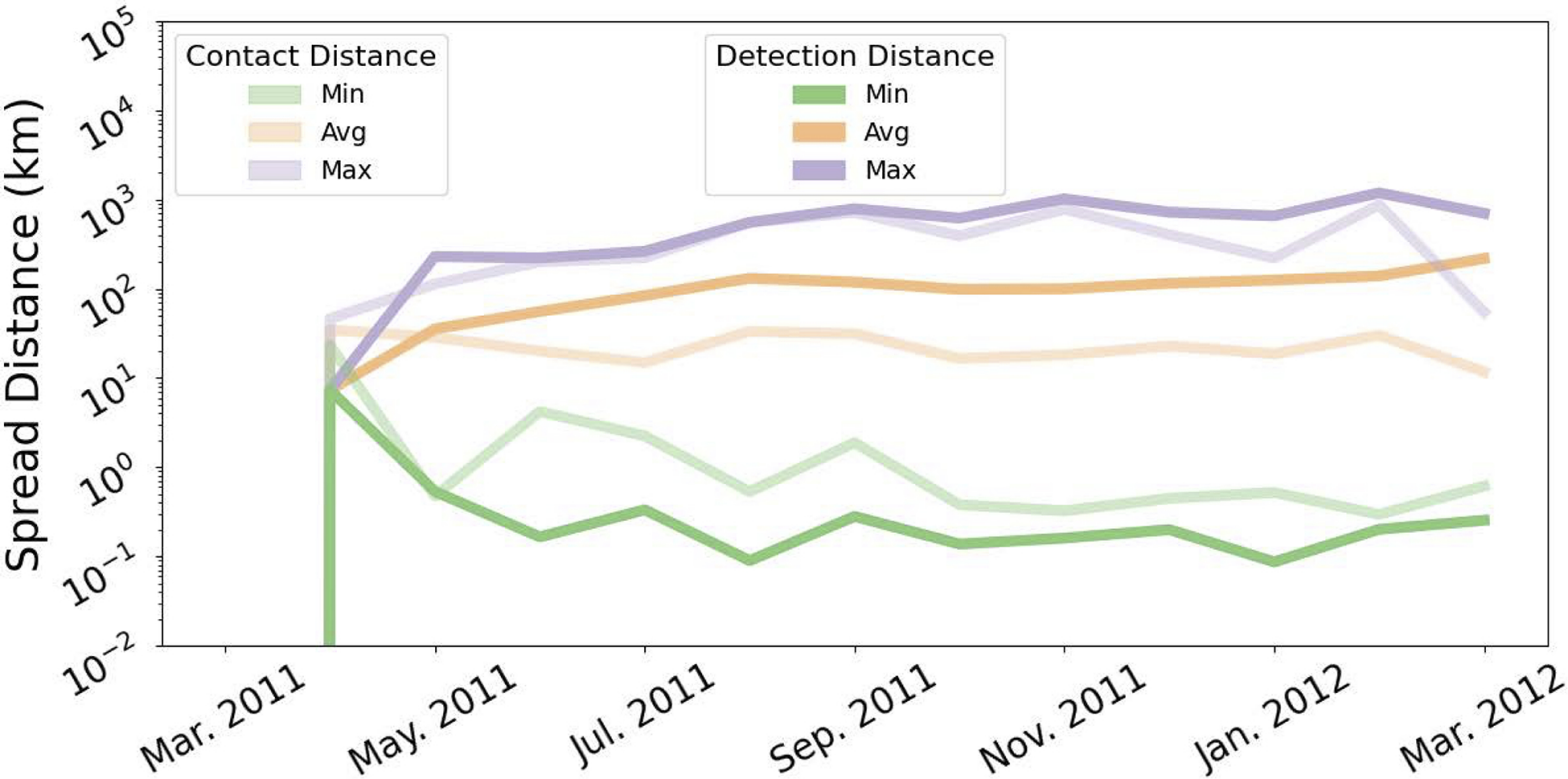
Spatio-temporal characterization of the spreading. Two distance metrics are compared using three different statistics: the mean, minimum and maximum. The detection-distance metric is calculated by comparing the distances of all properties that are infected within the same month. The contact distance is calculated by finding the average distance of a newly infected property's contacts. A contact is defined as a previously infected property that has an outgoing link to the newly infected property. We define a newly infected property as one that is detected as infected, not simply if it can transmit infection.

To compare the spread distance in our analysis with the potential spread distances for wind-related dispersal, we used wind-based matrices to estimate the time for a potentially wind-transported pathogen to travel from an infectious orchard (supplementary material). We observed that the wind transmission of Psa-V to connections within 20 km would take under 24 hours in most cases, which is comparable to that of our human-mediated model. However, longer-distance wind dispersal (>5 days) is much less probable, as it would require much longer travel time, often exceeding 5 days. Prolonged airborne survival requires consistently favourable environmental conditions that are less likely over longer periods. These observations are consistent with the literature; wind-related pathways are more likely to contribute to local spread than to long-distance spread for Psa-V. It is important to note that the within-property transmission probability in our model, $\beta_{w}$, is already incorporating this short-range environmental transmission, while between-property transmission can be extended in the future to incorporate short-distance environmental transmission upon the availability of more epidemiological data sufficient to avoid parameter identifiability issues.

### 3.3. Experimental analysis

#### 3.3.1. Detection threshold

The detection threshold D is the minimum fraction of infected property hectares for the property to be considered infected. The larger the value of D, the more hectares must become infected (see equation (2.6)). Figure 6 shows the temporal dynamics of the outbreak for different D values; we use the network constructed using seasonal network aggregation, and the rest of the model's parameters set to their best-fitting values (table 3, figure 4A). Decreasing D effectively allows us to observe outbreak occurrence earlier. Despite the peak incidence shifting by one month, the model illustrates similar temporal dynamics for different detection thresholds. The magnitude of incidence is similar between the smallest and largest values, and the final outbreak size only differs by 2.7%, decreasing from 56.1% of properties for D=0.2 to 53.4% for D=0.8. These results suggest that surveillance sensitivity alone is insufficient to reduce outbreak severity—implying that timely intervention following detection may be critical to altering final outbreak size and peak incidence.

**Figure 6.**
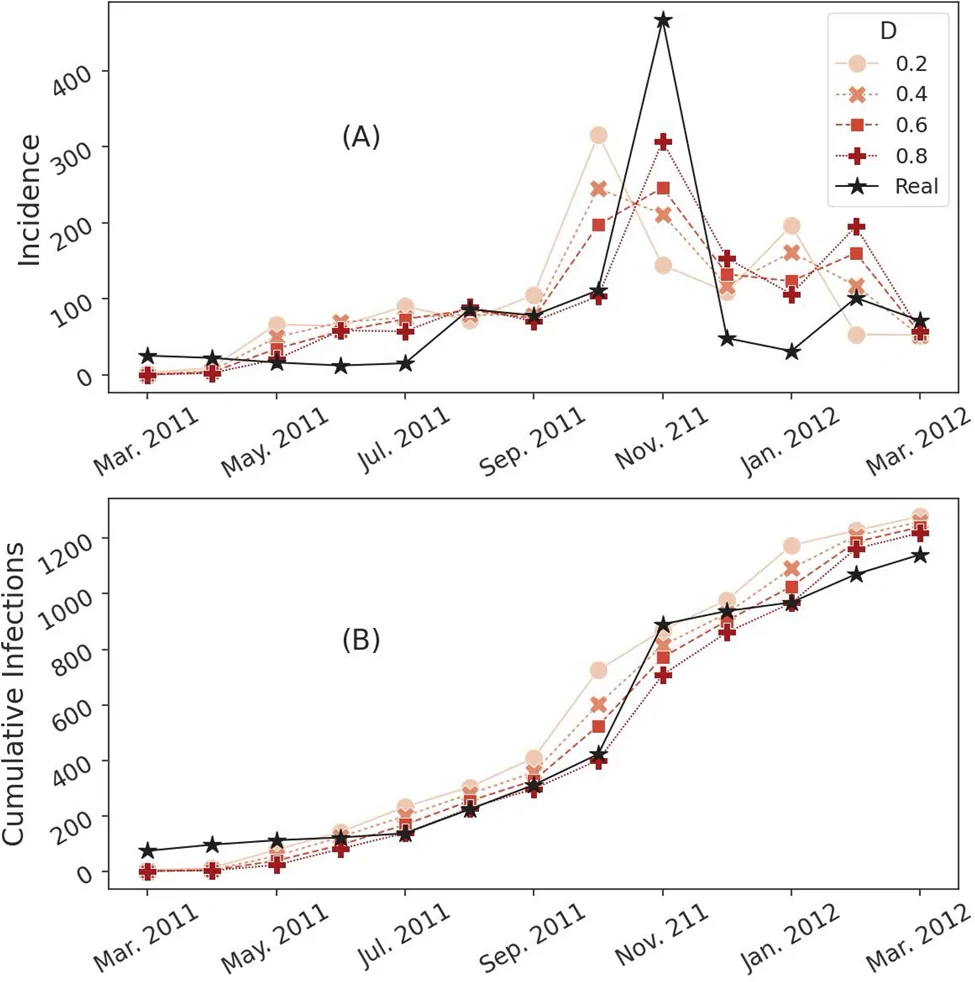
Dependence of the model's outcome on the detection threshold. We vary the detection threshold D and plot the effect this has on the incidence curve in (A) and on the cumulative total infections shown in (B). The best-fitting parameter configuration is D=0.8 for the seasonal network. In (A), we plot the raw incidence curve as a reference, and in (B) the raw cumulative infection data are plotted. For all graphs, the seasonal network is used as well as the corresponding parameters of the best fit (table 3).

#### 3.3.2. Importation month

All the above analyses were conducted using the assumption that the importation month was November 2010. Now, we consider hypothetical scenarios for a pathogen with the same epidemiological parameters where pathogen importation may occur at the beginning of each calendar month and at any seed property. For each month, we integrate the equations using the best-fitting estimates of the epidemiological parameters for the monthly aggregation (see table 3). To make the various scenarios comparable, the equations are integrated for the same 14 months. When the hypothetical importation month is set to November, we obtain very similar initial conditions to those used in previous results, with one key caveat—no filtering of the model estimates is being performed, and all seed properties within the Bay of Plenty are considered (either viable for observed outbreak or not).

A basic analysis of the constructed networks reveals that the volume of movements in the horticulture industry is highly seasonal, peaking in the spring and summer seasons (e.g. February and November in the inset of figure 7C) and reaching the lowest numbers in winter (June and July). This aligns with horticulture industry labour trends, where employment peaks in the summer and is smallest in the winter [56,57]. To evaluate the impact of the timing of pathogen importation on human-mediated transmission, we consider four different metrics: (i) the overall number of infections (figure 7A), (ii) the peak incidence (figure 7B), (iii) the time when such a peak is reached (figure 7D), and (iv) the effective start of the epidemic, i.e. the first instant of time when at least two properties are detected as infected (figure 7C). We show the number of total movements per month in the inset of figure 7C and compare the cumulative incidence trends against the number of movements per month. The variation in distributions correlates with the differences in the movement network associated with the different importation months.

**Figure 7.**
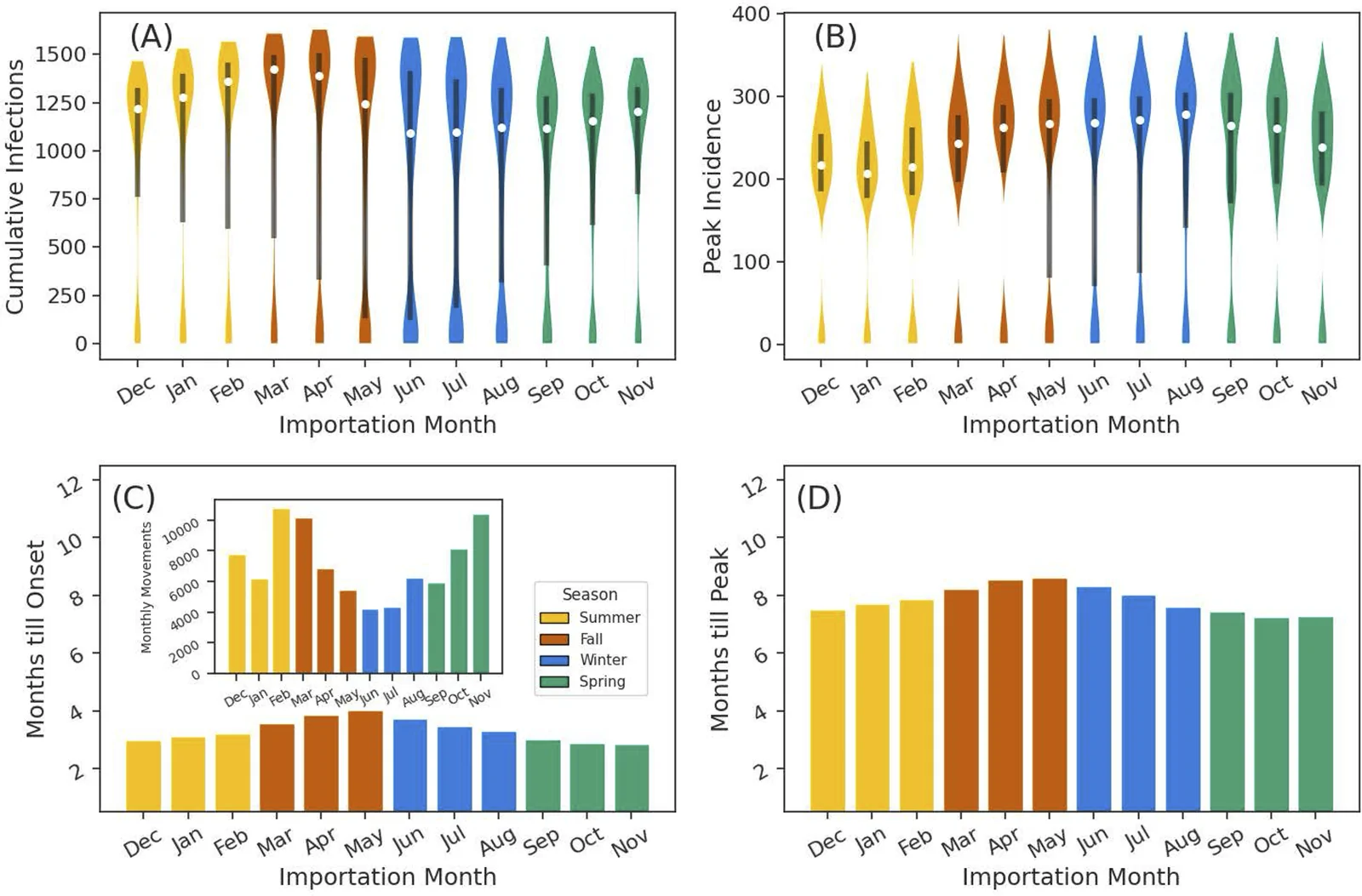
Dependence of the model's outcome on importation month. (A) The distribution of the final outbreak size (violin plots) for each importation month. Black boxes show the 50% interquartile range. White circles show the median value. (B) As in (A), but for the distribution of peak incidence. (C) The mean number of months from importation until the onset (time when two infected farms are detected). In the inset, the total number of movements per month is shown. (D) The mean number of months from importation until the month of peak incidence. Colours of bars and violins denote seasons.

Every month, there is a fraction of seeds where the final outbreak size is smaller than 250 infections. In May–July, this fraction increases together with an overall decrease in outbreak size heterogeneity, resulting in a smaller median final outbreak size. Our results show that the biggest outbreak sizes are observed when importation occurs in March and April, i.e. the months with the maximum number of movements.

We observe that importations in the autumn and winter months lead to a higher incidence at the peak of the outbreak, with the highest variability in May through August. There exists a consistently higher probability of the outbreak either not taking off or remaining very small in winter, reflected in similar trends for the final outbreak size and peak incidence. However, for the combination of epidemiological parameters and importation seeds that results in bigger outbreaks, peak incidence tends to be highest for winter importations and lowest for summer importations. With the median final outbreak size being the highest for fall importations and lowest for winter importations, this suggests different spatio-temporal dynamics for different seasons.

Finally, we see that the onset (two to four months) and peak (seven to eight months) times are almost constant irrespective of the importation month. We also find that it takes longer for outbreaks to peak (figure 7B) and start (figure 7C) if they begin in the autumn or winter seasons. These results come with the caveat that our model does not take into account temporal factors such as potential weather- and season-dependent susceptibilities of the plant to the infection and viability of the bacteria, which may all have an effect on the temporal outbreak trajectory.

## 4. Discussion

We use a metapopulation model that incorporates human mobility to simulate plant pathogen spread. Our model reproduces the timing and the dynamics of the Psa-V outbreak that occurred in 2010–2012 in New Zealand, demonstrating that human movement data can be effectively utilized to model the human-mediated spread of plant pathogens. This work is one of the first to use the dynamics of human mobility to simulate the human-mediated spatio-temporal spread of plant diseases. While other works have focused on how environmental factors [10,24], vectors [29,30] or farm proximity [43] affect pathogen dispersal, we show that the human movement network can affect both the size and speed of an outbreak when human-mediated transmission is epidemiologically relevant. Upon successful validation during future outbreaks, the proposed framework opens the door for utilizing human mobility networks for forecasting, scenario modelling and decision-making support for plant pathogens with human-mediated routes of transmission.

A key novelty of this work lies in providing a modelling framework to simulate both the short- and long-distance patterns of human-mediated dispersal of bacterial pathogens. Much of the existing literature [26,42,64] on the long-distance dispersal of plant diseases focuses on fungal pathogens, whose national or intercontinental spread is primarily driven by environmental factors like wind. For bacterial diseases, however, environmental factors such as wind-driven rain [53] and vectors [30] are generally considered major contributors to short-range dispersal, usually within 10 km for Psa-V [53,62], while human-mediated spread drives long-range dispersal [53,65]. In the intermediate range (e.g. 10–20 km) wind-driven dispersal can still contribute to transmission events together with the human-mediated factors. Our results suggest that when environmental between-property transmission is not explicitly simulated and in the absence of additional vectors such as insects or wind, human-mediated spread results in major local transmission, for Psa-V within 10 km. Furthermore, the human mobility connecting distant properties and regions allows us to simulate a rapidly expanding nationwide outbreak, driven by rare long-distance movements that cause sudden jumps in distance spread. Consequently, this study represents a crucial first step toward understanding how human mobility can affect the transmission dynamics of bacterial plant pathogens across multiple spatial scales when human-mediated dispersal is a viable transmission mechanism. However, the relative contribution of human-mediated versus environmental transmission will vary considerably across pathosystems [3,41], potentially leading to different outcomes for other pathogens.

Our calibrated model also allows us to test the sensitivity of human-mediated transmission to initial conditions. We find that the median size of an outbreak increases if the pathogen is imported during months with higher movement frequency. Holding the time of importation constant, the specific geographic location where the disease is seeded contributes to the large variability in epidemic severity. Some seed properties generate only small and local outbreaks, and others cause system-wide epidemic events.

Worker movements are intrinsically tied to the growing season, meaning that the density of movements per property changes based on seasonal agricultural demands. This highlights the importance of modelling how the movement network changes in time, since its temporal dynamics strongly affect the connectivity between properties and potential risks of human-mediated dispersal of pathogens. To explore the impact of temporal resolution, the proposed metapopulation model relies on underlying mobility networks aggregated over time windows of variable length (i.e. monthly, seasonal and yearly). While all of our network aggregations approximate the infection dynamics well, the seasonal network fits the raw data dynamics best owing to seasonal resolution capturing most of the changes in movement complexity. Understanding this seasonality and its drivers can be helpful to improve outbreak management and surveillance strategies. More generally, this modelling framework provides a foundation that, once recalibrated for a specific pathogen and region with appropriate epidemiological and movement network data, could potentially support short-term outbreak forecasting and scenario modelling—though prospective validation would be needed to confirm forecasting skill.

While our modelling approach can provide insights into the impact of human movements on the spread of plant diseases, our work has several limitations. First, our model has not been extended to account for the impact of environmental factors. For Psa-V, these environmental factors, specifically humidity, rainfall, wind-driven rain and temperature, have been found to contribute to local dispersal, pathogen survival and probability of observing symptoms for Psa-V [53,55]. Although Psa-V can overwinter [55] and survive for long periods of time in plants and soil, it is unknown how well it survives when transported through wind-related pathways. For Psa-V, unlike for fungal pathogens [25,26], the environmental factors are understood to contribute more to local spread and less to long-distance dispersal. These observations and our comparisons between wind-based matrices and our mobility network support our decision to focus on human mobility networks as the primary driver of long-range spread. Moreover, owing to parameter identifiability issues, these two modes of transmission—environmental and human-mediated—cannot be incorporated simultaneously without additional data. Accurate calibration of their relative contributions would require additional spatio-temporal epidemiological data to distinguish the environmental force of infection from the human-mediated route. By deliberately isolating the human-mobility component, our calibrated model avoids these identifiability constraints to provide a targeted assessment of long-range spread, even if it provides conservative estimates for short-range transmission between adjacent properties associated with potential environment-driven transmission.

Second, the observed epidemiological curves used to calibrate the model were affected by active interventions aimed at mitigating Psa-V spread [49,52]. The kiwifruit industry responded to the outbreak by establishing a surveillance and response system, implementing screening and washing of potential vectors (e.g. plant materials, vehicles) and providing guidance on best practices to the orchardists. In the most affected area of New Zealand (Te Puke in Bay of Plenty), up to 8% of the kiwifruit were cut out before the harvest in 2012 [52] (end of our calibrating window), and 51.8% of the production area was sprayed in 2011. However, owing to the unavailability of precise data on the deployed interventions across time and space, interventions were not explicitly modelled in this study.

Third, in this model, we used the Onside horticulture movement network to simulate the spread of a pathogen that primarily affects the kiwifruit industry. Two aspects of this choice warrant discussion. First, while Psa-V poses a severe threat to kiwifruit orchards, human-mediated transmission continues even if vectors (such as planting and pollinating materials, footwear, clothing or vehicles) pass through a non-kiwifruit property. Utilizing only the network of kiwifruit properties would underestimate true connectivity and, consequently, the potential outbreak size and dynamics. Therefore, the broader horticulture movement network was used to better capture transmission-relevant mobility. Second, while this mobility network does not provide absolute coverage of the horticulture industry—potentially affecting overall network size and connectivity—it represents a majority of the industry's properties in New Zealand. For example, the dataset covers 70% of the kiwifruit production area and spans all geographic regions where orchards exist. As industry adoption of this technology continues to grow, these data will provide an even more robust foundation for the proposed modelling framework in the future.

While our model captures well the timing of the peak and the peak incidence for the moving average data with the point estimate being only 5.53–16.6% lower than the observed data, it underestimates the magnitude of the raw peak incidence in November 2011 by 33.8–41.3% depending on the level of temporal aggregation. This difference in model behaviour may be explained by a set of factors. First, the reporting of the infected orchards may have increased over time as the surveillance system was established during the outbreak. Second, our model does not consider the fluctuations in the environmental factors, such as rainfall and temperature, that may have caused a sudden increase in the symptoms and/or transmissibility in spring. Third, as interventions were not explicitly considered in the model, our analysis does not consider their potential temporal variation, which may have contributed to a sharp increase/decrease in the number of reported infected orchards. However, the modelling approach is flexible enough to allow for the incorporation of these aspects in future work, provided that suitable epidemiological data are available. As the model can better fit the moving average data as the temporal resolution of the network data improves, this may also indicate that more granular temporal data are needed to better capture changes in transmission dynamics. We also suggest that any use of this framework for biosecurity decision-making should incorporate uncertainty estimates for peak estimates to avoid underestimating response needs.

While this study effectively illustrates the calibration pipeline using a straightforward filtering and RMSE approach, the framework is designed to be adaptable, allowing future research to incorporate traditional inference methods, such as Markov-chain Monte Carlo methods [66] or particle filtering [67]. This work could also be extended to incorporate: (i) environmental layer for between-property localized dispersal, (ii) environment-sensitive incubation period by introducing an exposed compartment, and (iii) interventions, such as quarantine, spraying and trade/movement reduction by introducing removed compartments. This, however, would require detailed surveillance data on the presence of the pathogen and interventions at the appropriate spatial and temporal resolution.

The modelling framework developed here, combined with the Onside movement network, could be recalibrated using surveillance data for a novel bacterial plant disease spreading in New Zealand, providing a data-driven tool suitable for testing potential outbreak scenarios upon successful validation. This modelling approach also offers several promising avenues for future research, including identifying effective organization of sentinel nodes for surveillance systems [45,68] to ensure the timely detection of outbreaks and evaluation of various containment strategies to mitigate disease spread once an outbreak is identified.

## Supplementary Material



## Data Availability

All the code and data needed to reproduce the results of the study are available on Zenodo at [69] and GitHub at http://github.com/vakrao/net_plant_spread. To prevent unauthorized public disclosure and protect the identifiability of individual properties and their owners, granular location data (latitude and longitude of each property) will not be released publicly owing to legal and privacy restrictions. These proprietary data are subject to the Onside Privacy Policy, Terms of Use and the New Zealand Privacy Act 2020. Researchers interested in accessing the raw, granular data for reproduction purposes may contact Onside at info@getonside.com Supplementary material is available online [70].
