# Supplementary material for: Modelling plant disease spread via high-resolution human mobility networks
Source: J R Soc Interface. 2026 Oct 7;23(243):20260213. doi: 10.1098/rsif.2026.0213 (PMC13641950; doi:10.1098/rsif.2026.0213)
Supplement: Supplementary file 1 [file rsif.2026.0213.zip › rsif.2026.0213/rsif20260213_si_001.pdf]

rsif.royalsocietypublishing.org

Research

Article accepted for publication in Journal of The Royal Society Interface.
DOI: 10.1098/rsif.2026.0213

Subject Areas:
Computational biology, Biocomplexity

Keywords:
mathematical modeling, networks, mobility network, plant disease, disease spread, biosecurity

Author for correspondence:
Maria Litvinova
e-mail: malitv@iu.edu

Supplementary Materials: Modeling plant disease spread via high-resolution human mobility networks

Varun K. Rao[1,2], Ryan Higgs[3], Hautahi Kingi[3], Filippo Radicchi[1], Santo Fortunato[1], Maria Litvinova[2]

[1]Center for Complex Networks and Systems Research, Luddy School of Informatics, Computing, and Engineering, Indiana University, Bloomington, Indiana 47408, USA
[2]Department of Epidemiology and Biostatistics, School of Public Health - Bloomington, Indiana University, Bloomington, Indiana 47408, USA
[3]Onside, Christchurch, New Zealand

© The Authors. Published by the Royal Society under the terms of the Creative Commons Attribution License http://creativecommons.org/licenses/by/4.0/, which permits unrestricted use, provided the original author and source are credited.

1. Numerical Integration of the Mean-Field Approximation

Denote with $I_i(t)$ the fraction of hectares that are infected in farm i at time t, and with $S_i(t)$ the fraction of hectares that are not infected in farm i at time t. We clearly have $I_i(t) + S_i(t) = 1$ for all $i = 1, \ldots, N$ and all $t \geq 0$.

The farm-based mean-field equations for the SI dynamics are

$$\frac{d\,I_i(t)}{dt} = \beta_{\mathrm{w}}\, I_i(t) S_i(t)\, \Theta\left[I_i(t) - I_i^{\mathrm{min}}\right] + \beta_{\mathrm{b}}\, S_i(t)\, \sum_{j=1}^{N} w_{j\to i}(t)\, I_j(t)\, \Theta\left[I_j(t) - I_j^{\mathrm{min}}\right] \quad , \tag{S1}$$

where β_w and β_b are the transmission rates within and between farms, respectively. $\Theta(x)$ is the Heavyside function defined such that $\Theta(x) = 1$ if $x > 0$ and $\Theta(x) = 0$ otherwise. To be considered infected and contribute to the spreading dynamics, property i should have a level of infection that is above the threshold $I_i^{\mathrm{min}} = \left(H_i\, 625\,\text{plants hect}^{-1}\right)^{-1}$, where 625 is a realistic estimate of the average number of plants per hectare [1]. $w_{j\to i}$ is proportional to the number movements between properties j and i observed at time t.

We can also write the above as a finite-difference equation

$$I_i(t+dt) = I_i(t) + \beta_w\, dt\; I_i(t)S_i(t)\Theta\left[I_i(t) - I_i^{\mathrm{min}}\right] + \beta_b\, dt\; S_i(t) \sum_{j=1}^{N} w_{j\to i}(t)\, I_j(t)\, \Theta\left[I_j(t) - I_j^{\mathrm{min}}\right] \tag{S2}$$

To perform the numerical integration, we require that

$$\beta_w\, dt\; I_i(t)S_i(t)\Theta\left[I_i(t) - I_i^{\mathrm{min}}\right] + \beta_b\, dt\; S_i(t) \sum_{j=1}^{N} w_{j\to i}(t)\, I_j(t)\, \Theta\left[I_j(t) - I_j^{\mathrm{min}}\right] \leq 1 - I_i(t)\,. \tag{S3}$$

leading to the constraint for the time increment:

$$dt \leq \frac{1 - I_i(t)}{\beta_w I_i(t)S_i(t)\Theta\left[I_i(t) - I_i^{\mathrm{min}}\right] + \beta_b\; S_i(t)\; \sum_{j=1}^{N} w_{j\to i}(t)\, I_j(t)\, \Theta\left[I_j(t) - I_j^{\mathrm{min}}\right]}\,. \tag{S4}$$

Such a condition means that Eq. (S2) can effectively be used to numerically integrate Eq. (S1). Since the above must be true for all farms, we need to impose

$$dt \leq \min\left(T_{\mathrm{inc}}, \min_{i=1,\ldots,N} \frac{1 - I_i(t)}{\beta_w I_i(t)S_i(t)\Theta\left[I_i(t) - I_i^{\mathrm{min}}\right] + \beta_b\; S_i(t)\; \sum_{j=1}^{N} w_{j\to i}(t)\, I_j(t)\, \Theta\left[I_j(t) - I_j^{\mathrm{min}}\right]}\right). \tag{S5}$$

where $T_{\mathrm{inc}} = 1$ to generate at least one data point per day.

2. Infection Data

The number of monthly cumulative infections and incidence from the 2010 Psa-V outbreak in New Zealand was digitized from Greer and Saunders [2] and is listed in Table S1. Raw data values are shown as well as the values for the trailing 2-month moving average. Using this incidence data we calibrated our model.

Table S1. Monthly raw counts and two-month moving average (MA(2)) incidence and cumulative infections.

Month	Raw Counts		2 Month Moving Average	
	Incidence	Cumulative	Incidence	Cumulative
Feb. 2011	50	50	50	50
Mar. 2011	25	75	25	75
Apr. 2011	22	97	23.5	98.5
May. 2011	16	113	19	117.5
Jun. 2011	10	123	13	130.5
Jul. 2011	15	138	12.5	143
Aug. 2011	86	224	50.5	193.5
Sep. 2011	88	312	87	280.5
Oct. 2011	111	423	99.5	380
Nov. 2011	467	890	289	669
Dec. 2011	48	938	257.5	926.5
Jan. 2012	31	969	39.5	966
Feb. 2012	101	1070	66	1032
Mar. 2012	71	1141	86	1118

3. Gridsearch Calibration

The model contains four free parameters which require calibration: the within-property spreading rate β_w, the between-property spreading rate β_b, the detection threshold D, and the initial seed property s_0. We test $1,300$ different seed properties located in the Bay of Plenty region and test several different values of the detection threshold D, where set $D = \{0.2, 0.4, 0.6, 0.8\}$.

To find an appropriate range of values for β_w and β_b, we first conducted a coarse exploratory gridsearch using logarithmic increments and searched over $[10^{-3}, 10^{0}]$. For each pair of dynamical parameters, (β_w, β_b), the average RMSE was calculated using all values of D and s_0. The RMSE landscape shown in Figure S1 indicates that the best parameter configurations are between $[10^{-2}, 10^{-1}]$ for all combinations of β_w, β_b, taking into account different data types and temporal aggregations. Informed by this finding, we performed a finer-grained gridsearch varying β_w, β_b (Figure S2 over the range $[10^{-2}, 10^{-1}]$ using increments of 0.01 across the set values of D and s_0 .

$$|x_c - y_c(\theta)| < \max\{k, b * x_c\} \tag{S1}$$

The average RMSE values shown in both gridsearches (Fig S1 and Fig S2) were calculated across the set of configurations G, consisting of all possible combinations of β_b, β_w, D, s_0 that were discussed for each gridsearch. Given G, and each configuration entry θ for all free parameters, we filtered configurations using the heuristic defined in the main text to prevent overfitting to the incidence curves.

We slightly redefine the filtering heuristic in Equation S1, using k and b as the two main variables that need to be set when performing the filtering. k is utilized as a tolerance value while b is the size of our uncertainty band. In the main text, $k = 30$ and $b = 0.8$. A gridsearch was conducted to find the best values for k and x using the raw data in Figure S3. k was varied from 10^1 - 10^2 in increments of 10^1 while b was varied from $10^{-1} - 1$ in increments of 10^{-1}. The percentage of viable configurations that existed by fulfilling the conditions in Equation S1 was recorded for each combination. From that gridsearch, $k = 30$ and $b = 0.8$ are the first two values that produce more than 1000 viable configuration among all aggregations, allowing for a variety of different configurations without overfitting to the real data. See the main text for details on how we selected the best configuration for each aggregation type.

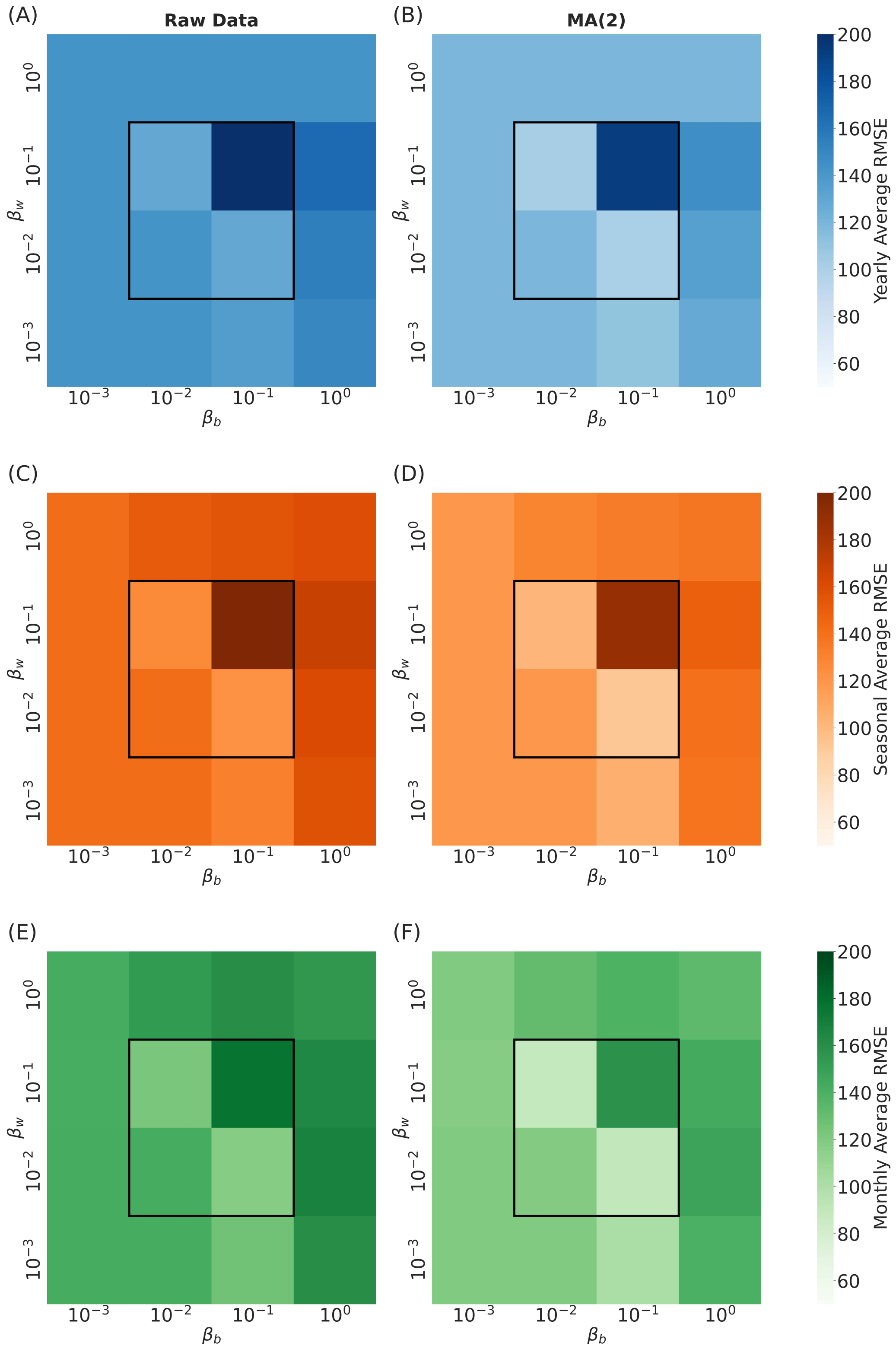

Figure S1. Logarithmic gridsearch calibration of β_b, β_w We present the average RMSE for the various combinations of (β_w, β_b) where we vary the values for these dynamical parameters from 10^{-3} to 10^{0} in logarithmic increments. Each square in the heatmap is the expected RMSE value for the joint distribution of β_w and β_b over all the possible s_0 and D combinations. The black square within each heatmap represent the values for β_b, β_w that were searched for in more depth in the linear gridsearch used in the main text. The RMSE values in the first column of heatmaps is calculated with reference to the raw data, while the values in the second column are calculated with respect to the two month moving average (MA(2)) data. In panel (A) we plot the RMSE for the yearly aggregation using the real data , in panel (B) we plot the average RMSE for the yearly network aggregation using the MA(2) data, in panel (C) we plot the average RMSE for the seasonal network aggregation using the raw data. while in panel (D) we plot the average RMSE for the seasonal aggregation using the MA(2) data. In panel(D) and (F) we plot the average RMSE for the monthly aggregation using the raw incidence data and MA(2) incidence data respectively .

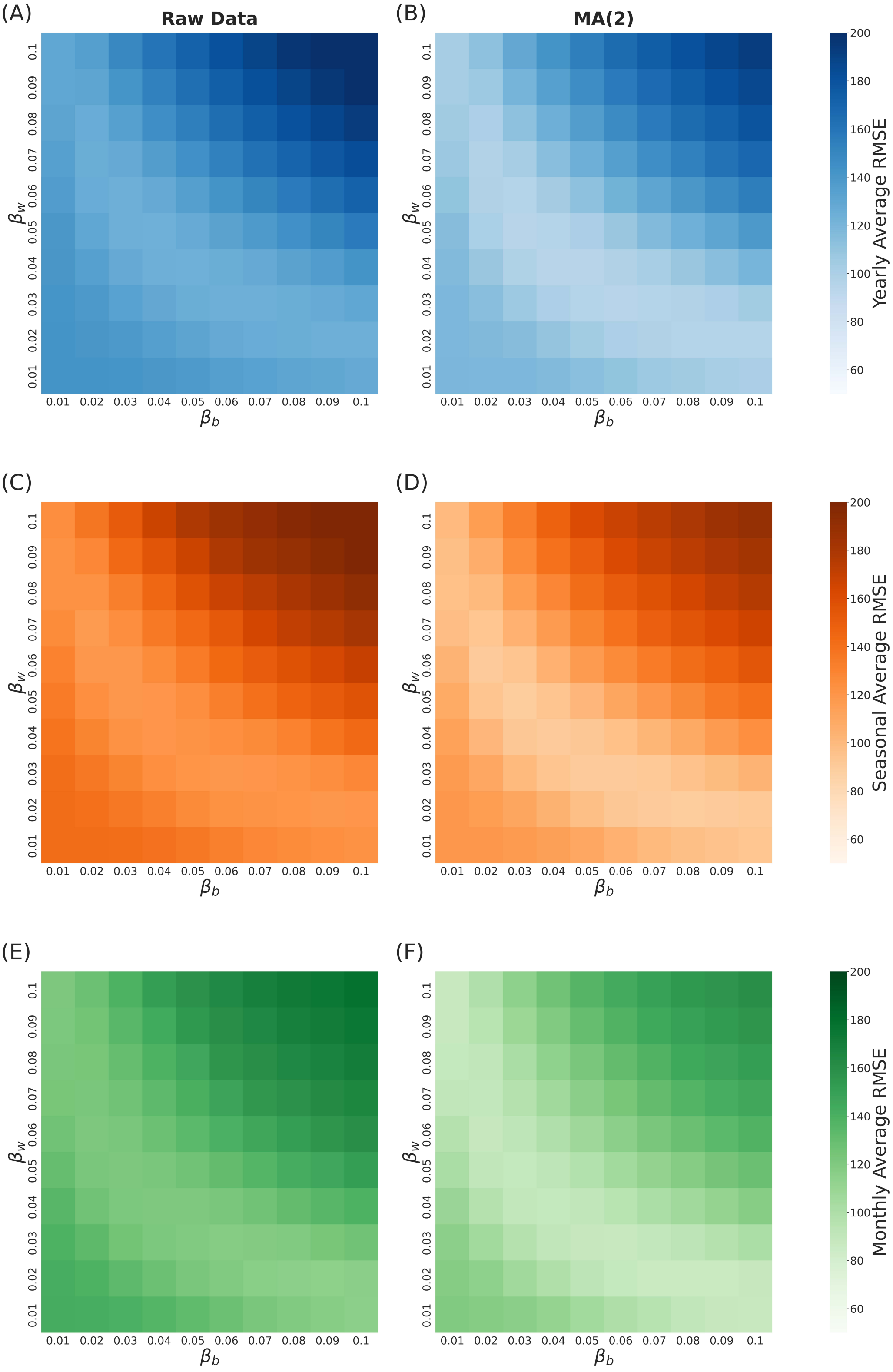

Figure S2. Linear gridsearch calibration of β_b, β_w Similar to Figure S1 we plot the average RMSE for combinations of of (β_w, β_b), where RMSE values in the first columns of the heatmaps are calculated with reference to the raw data, while the values in the second column are calculated with reference to the MA(2) data. We vary the values for these dynamical parameters from 10^{-2} to 10^{-1} respectively, in increments of 10^{-1}.

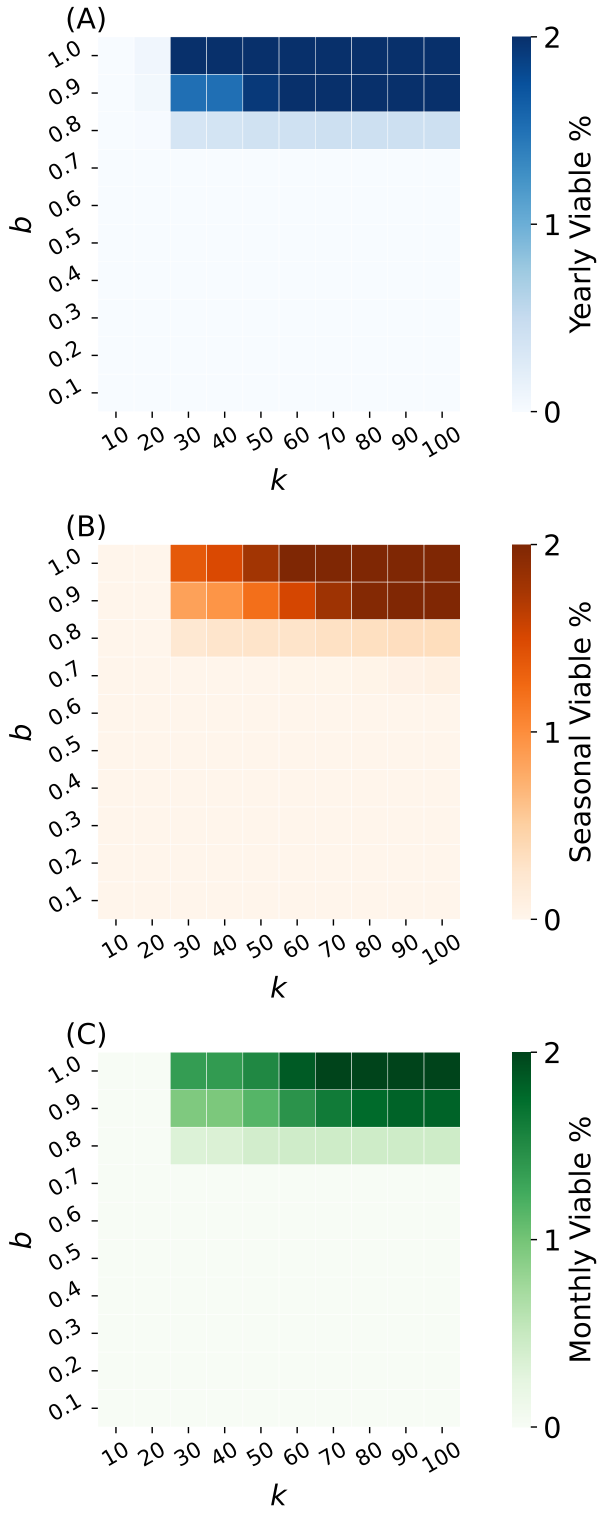

Figure S3. Parameter gridsearch for k and b. The parameter values for k and b are varied in this figure. We show the results of three different gridsearches, for all aggregations, using the raw incidence data to determine the percentage of viable configurations for each temporal aggregation. The darker the square in the heatmap, the higher percentage of viable configurations exist for that specified combination of k and b. Panel (A) reports the percentage of viable configurations for the yearly aggregation, (B) and (C) are the same as (A) except for the seasonal and monthly aggregations respectively.

Using the viable configurations for each aggregation and data type, we plot the posterior distribution of the parameters in Figure S4. We observe that there are strongly preferred values for all parameters and aggregations, barring the monthly aggregation for the raw data. For β_w and β_b, specific values are clearly preferred. For the monthly raw aggregation, there is a bimodal distribution.

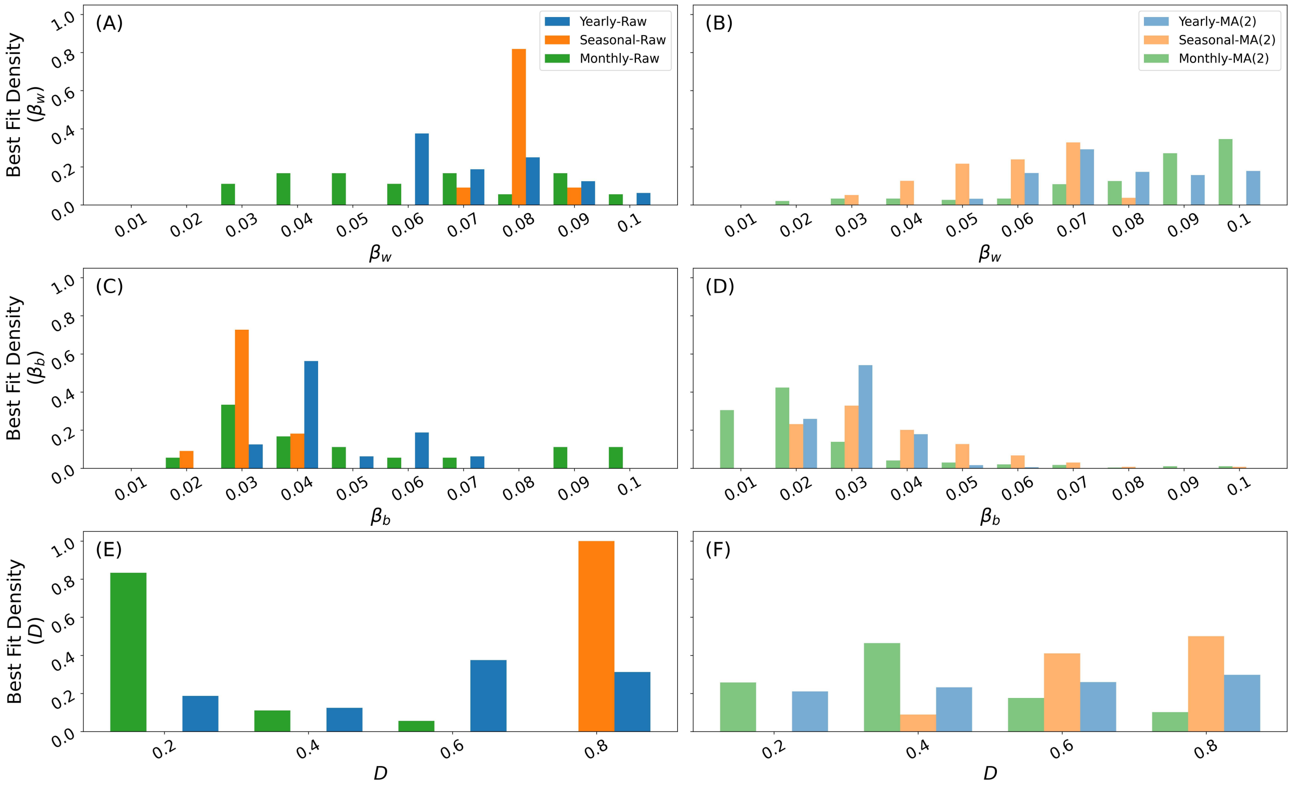

Figure S4. Posterior Distribution of Linear Gridsearch Using the RMSE values generated from the linear gridsearch, seen in Fig. S2, we plot the posterior distribution for each parameter (except the seed, due to the number of seeds). These distributions are generated after creating the viable configuration set V for each aggregation and data type (Raw and MA(2) incidence data) and limiting the viable configurations to the configurations that have the smallest 1% of RMSE values among all configurations V. The best-fitting configurations are found from the configurations in V that minimize the RMSE best for each configuration and dat type. The first columns RMSE values are calculated with reference to the raw data, while the second columns RMSE values are calculated with reference to the MA(2) data. Three different colors are used in each panel to reference different network aggregations. Blue represents the yearly aggregation, orange represents the seasonal aggregation, and green represents the monthly aggregation. Each row contains the posterior distribution for a different parameter. Panels (A) and (B) plot the distribution for β_w, panels (C) and (D) plot the distribution for β_b, and panels (E) and (F) plot the distribution for D. The RMSE values in panels (A), (C) and (E) are computed with respect to the raw data while in panels (B),(D), and (E) they are calculated using the MA(2) data.

4. Spatial Spread

The complementary cumulative distribution function (CCDF) of movement length (i.e., distance between origin and destination property) is calculated and shown in Fig. S5. 99.1% of movements occur between properties that are between $0-100$ km from each other, with 71.2 % of movements occurring having a distance of 10 km. From such a movement distribution, we can conclude that most movements in horticulture movement network occur between properties that are relatively close to one another. This movement structure leads to the results in Figure S6 where newly infected properties are infected within 10 km of another previously infected property. While most movements are short-distance, long distance spread from the seed still occurs, as we find in Figure S7, where by the end of the outbreak the epidemic has spread more than $1,000$ km from where it started.

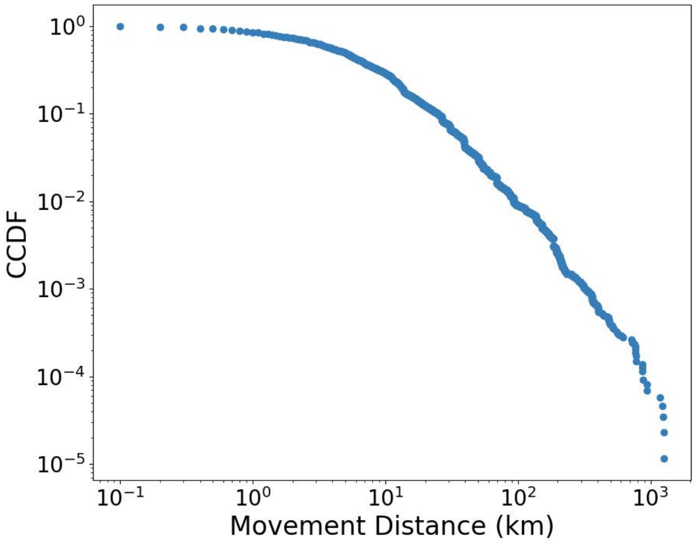

Figure S5. Complementary Cumulative Distribution Function (CCDF) of Movement Length.

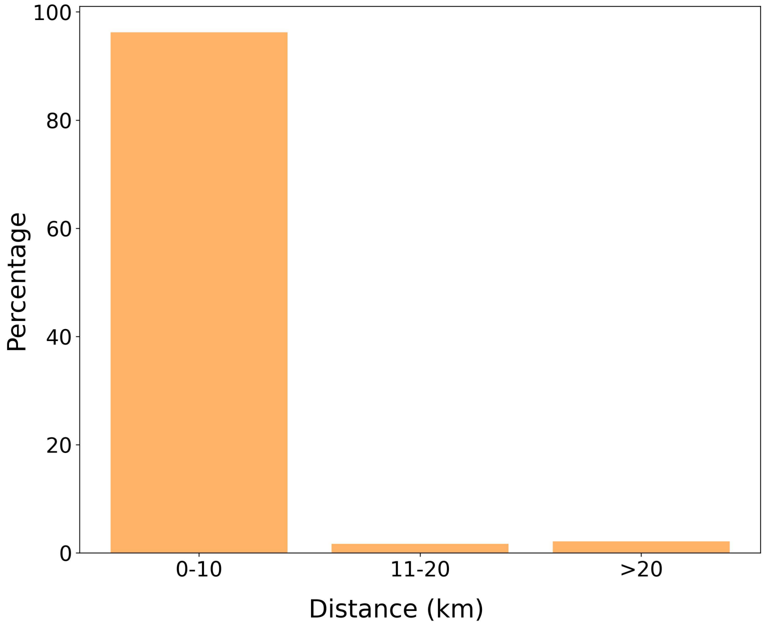

Figure S6. Distance of Newly Infected Property to the Closest Infected Property Once a property becomes infected, we measure the distance in kilometers from that property to the closest infected property. An infected property i is defined as one where the level of infection on it exceeds D where $I_i > D$. We classify this distance using three categories: $0-10$ km, $11-20$ km, and > 20 km. Most newly infected properties are within $0-10$ km of a previously infected property, and only 2.7% of properties are > 20 km away from an infected property.

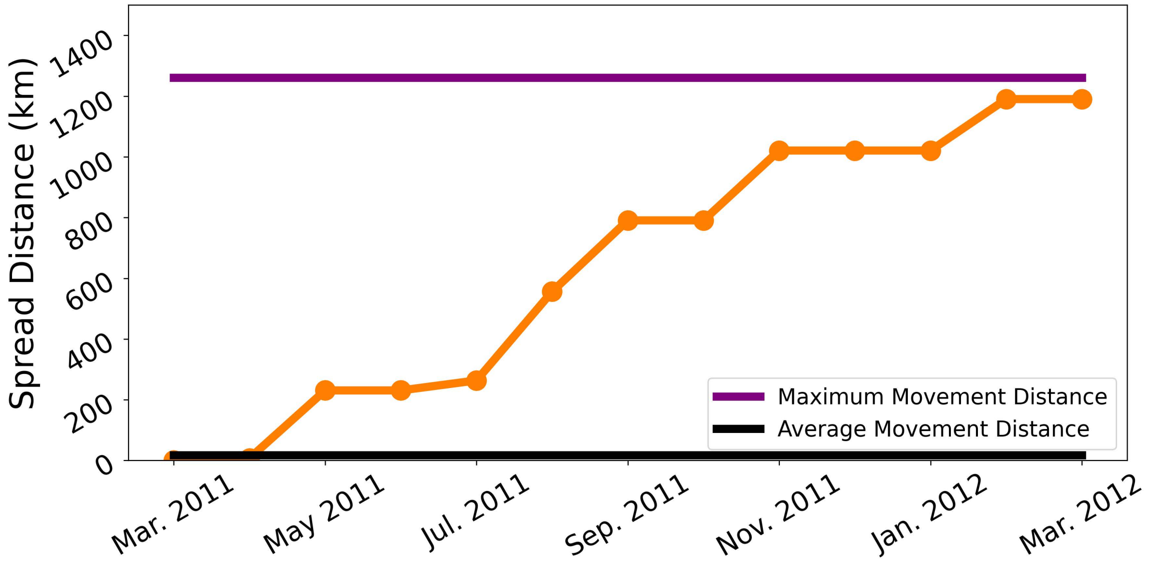

Figure S7. Maximum Spread Distance from Seed Using the calibrated seasonal model, we plot the largest geodesic distance an infected property has from the initially infected seed property s_0. We define this distance as the spread distance. The node with the largest distance away from the seed could persist for months, as we observe that for consecutive months at a time the spread distance does not change in value. As reference, we plot the maximum and average movement distance between any two properties as well. By the end of the outbreak, the distance from the seed is very close to the maximum existing movement distance.

5. Wind Connectivity Matrix

Leveraging wind reanalysis data spanning the years 2010-2012 from the ERA5 [3] dataset we utilize the R package windscape [4] to generate a wind connectivity matrix for New Zealand. The ERA5 dataset compiles hourly high-resolution data over a latitude-longitude grid with a resolution of roughly 31 km. Using this matrix, we can estimate the wind travel time in hours between any two locations in New Zealand. First, we plot the wind-interconnectivity matrix in Figure S8 over a map of New Zealand, where we show a subset of nodes from our yearly mobility network. We selected a node i (in red) with the largest number of connections j (nodes with at least one movement between i and j, in white). The majority of these connections are within 20 hours of travel time from i (assuming favorable conditions), but a few exceed that value. The wind conditions do not inhibit spreading; however, the observed patterns would make long-distance dispersal difficult in this example, as it would require more than 60 hours of pathogen survival and consistent favorable conditions. For privacy reasons, the latitude and longitude values are not shown.

Using the wind-connectivity matrix, which we partially graph in Figure S8, we plot the relationship between wind travel time and distance between all i in the yearly network and all of their connections in Figure S9. We observe a strong, positive linear relationship between the two in Figure S9, showing that as the distance between two properties grows, the travel time required for the transmission increases. These results show that while it requires less than a day for a pathogen to be transmitted through human mobility connections, it may require more than 10 days for the wind-based dispersal to reach the same location. This highlights the higher importance of incorporating human mobility networks for better understanding human-mediated long-distance dispersal.

The CCDF (Complementary Cumulative Distribution Function) in Figure S10 shows that when the distance between a property and its connections exceeds 20 km, there is a higher probability of the required travel time to exceed one day. These two figures highlight how adding dispersal via wind would lead to more spread within properties and between close properties.

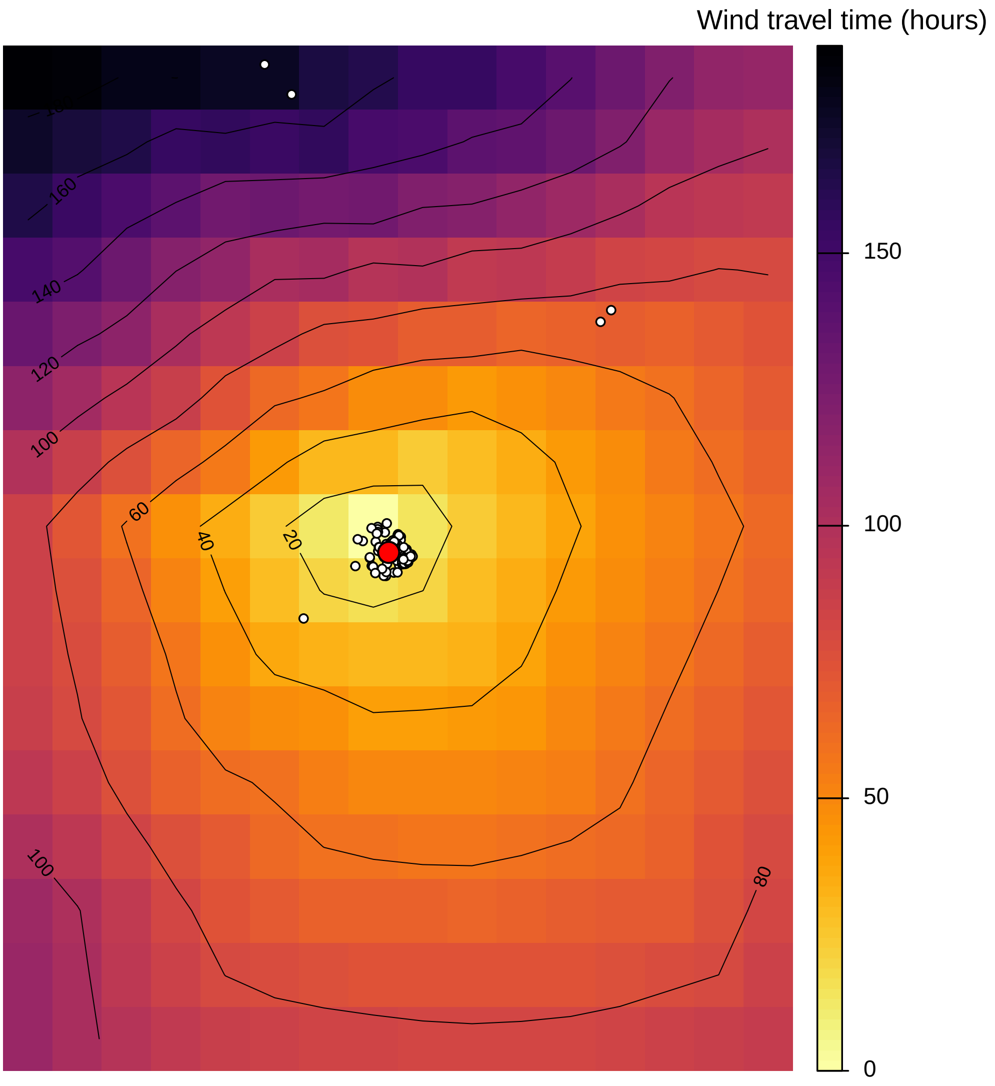

Figure S8. Windmap Example Wind connectivity matrix between a highly-connected network node and all of its connections. The raster map color shows the amount of time for a wind particle to travel. The node is shown in red, its connections are shown in white.

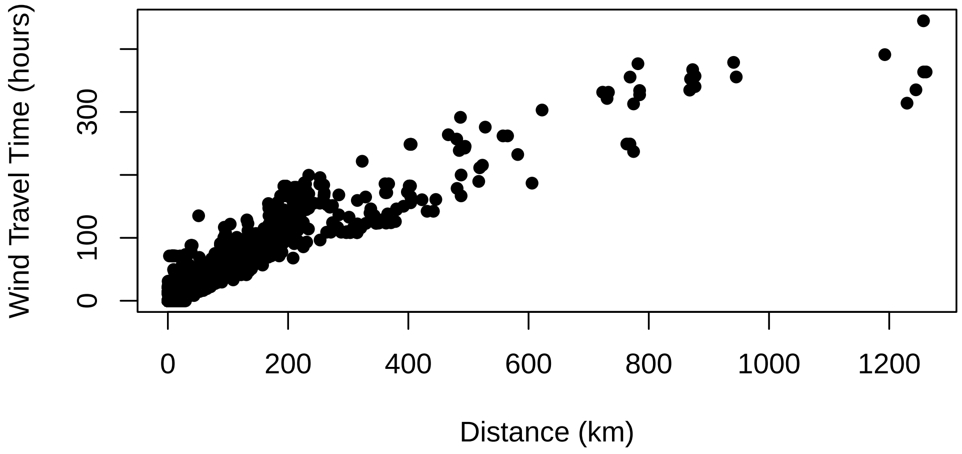

Figure S9. Relationship between Wind Travel Time and Distance Every node has a set of connections. This figure illustrates the relationship of the physical geodesic distance between a node and its connections and the wind travel time potentially needed for a wind-based dispersal to occur.

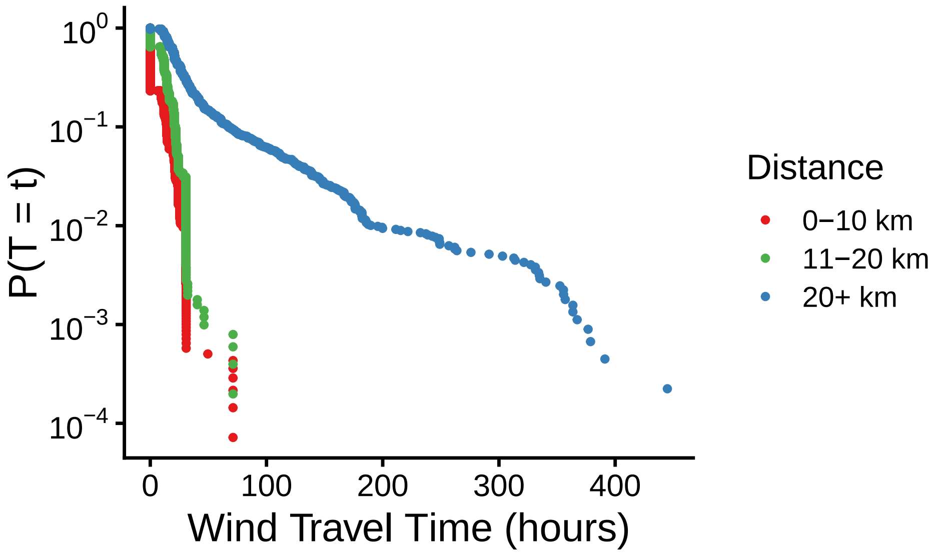

Figure S10. CCDF of Wind Travel The CCDF shows the wind travel time by distance class. Red dots refer to a property's connections within 10km, green dots refer to a property's connections 11-20km from it, while blue refers to a property's connections further than 20km from it. The y-axis denotes the probability of observing a value of the wind travel time t greater than the listed y-value T.

6. Comparing the Best Fits Based on Temporal Network Aggregations

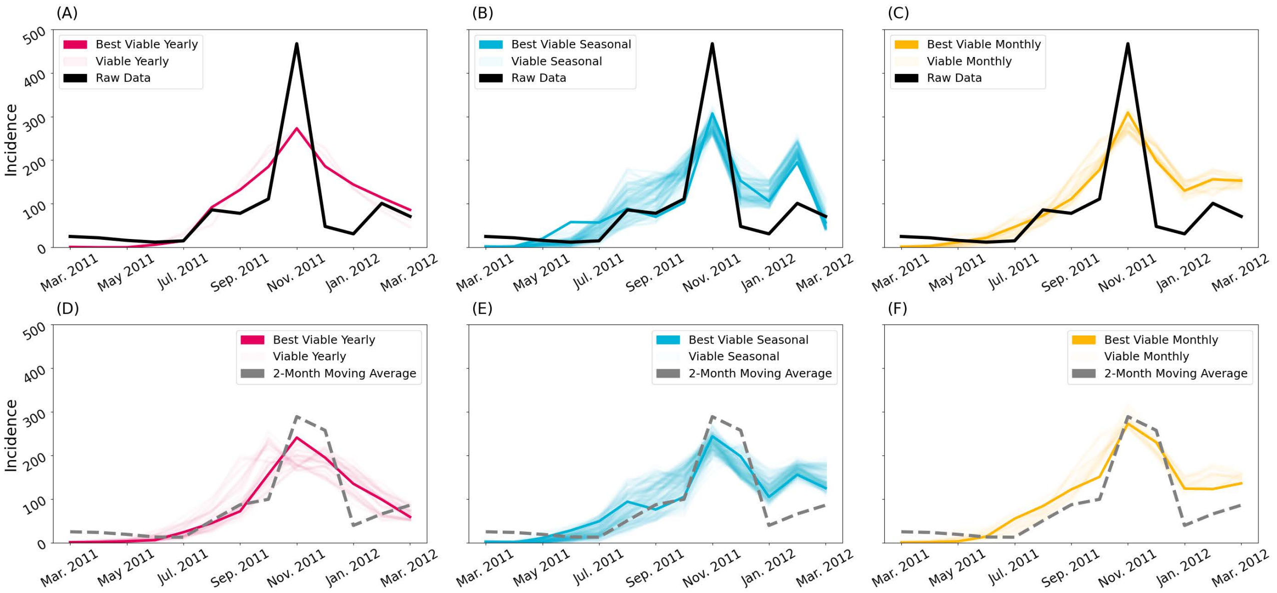

Figure S11. Model fit for different network aggregations Each panel shows the best-fitting configuration of each aggregation in a solid color. The lines in lighter/transparent color represent the other possible viable configurations with the same values for β_b, β_w, D as the best-fitting configuration but varying seed locations s_0. The first row of figures shows fits for the raw data, while the second row shows fits for the moving average data. Panels (A) and (D) show yearly aggregations for the raw and moving average data, respectively. Panels (B) and (E) show seasonal aggregation for the raw and moving average data, respectively. Panels (C) and (F) show monthly aggregation for the raw and moving average data, respectively.

In Figure 4 of the main text, we show the best-fitting configurations along with uncertainty bands generated based on viable parameter configurations. For each aggregation, we selected a best-fitting parameter configuration for β_w, β_b, D based on our calibration procedure, and simulated the outbreak for each s_0. Therefore, the uncertainty band represents the variation of the trajectories due to the selection of different seed properties.

In Figure S11, we show the individual trajectories of these simulations in lighter colors and the best-fit parameter selection in darker colors for each network aggregation, providing a more detailed variation of Figure 4. This figure also highlights the differences in the model fit for the raw data and the moving average. For the moving average dataset, we observe how the graph better represents the peak as the network aggregation resolution changes from yearly to seasonal to monthly.

7. Varying Initial Infection Ratio

We calibrate the model assuming that the seed property's (s_0) infection level (I_{s_0}) is set to 0.1. Figure S12 shows the temporal dynamics of the outbreak when the initial amount we infect the seed with, I_{s_0}, is varied. We denote I_{s_0} as α for this experiment. We vary α using the calibrated model based on the seasonal network. When calibrating the model $\alpha = 0.1$, we find that increasing α increases the size of the outbreak while not having a large effect on the incidence dynamics. This result highlights the small effect that α has on outbreak size, and its minimal effect on outbreak dynamics.

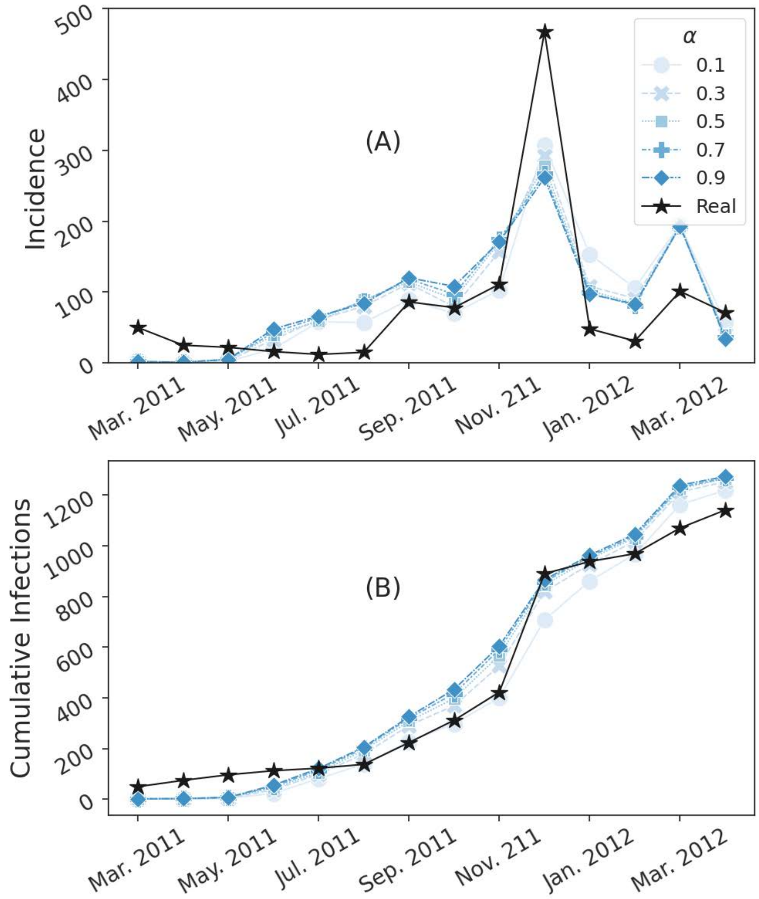

Figure S12. Dependence of Model's Outcome on Initial Seed Value We vary the initial seed value α and plot the effect this has on the incidence curve in panel (A) and on the cumulative total infections shown in panel (B). We set $\alpha = 0.1$ when calibrating the model. Here we vary α from 0.1 to 0.9 in increments of 0.2 in both panels. In panel (A) we plot the real incidence curve as a reference, and in panel (B) the real cumulative infection data is plotted. For all graphs, the seasonal network is used as well as the corresponding parameters of best fit

References

[1] New Zealand Kiwifruit Growers Inc., *New Zealand Kiwifruit Book 2024*, Accessed: 2025-04-08 (New Zealand Kiwifruit Growers Inc., 2024).

[2] G. Greer and C. M. Saunders, “The costs of Psa-V to the New Zealand kiwifruit industry and the wider community”, (2012).

[3] H. Hersbach et al., *ERA5 hourly data on single levels from 1940 to present*, Accessed: 20-05-2026 (Copernicus Climate Change Service (C3S) Climate Data Store (CDS), 2023).

[4] M. M. Kling and D. D. Ackerly, “Global wind patterns shape genetic differentiation, asymmetric gene flow, and genetic diversity in trees”, Proceedings of the National Academy of Sciences **118**, e2017317118 (2021).
